# Influence of formic acid treatment on the proteome of the ectoparasite *Varroa destructor*

**DOI:** 10.1371/journal.pone.0258845

**Published:** 2021-10-26

**Authors:** Antonia Genath, Hannes Petruschke, Martin von Bergen, Ralf Einspanier

**Affiliations:** 1 Institute of Veterinary Biochemistry, Freie Universität Berlin, Berlin, Germany; 2 Department of Molecular Systems Biology, Helmholtz Centre for Environmental Research-UFZ, Leipzig, Germany; 3 Faculty of Biosciences, Institute of Biochemistry, Pharmacy and Psychology, University of Leipzig, Germany; University of Alberta, CANADA

## Abstract

The ectoparasite *Varroa destructor Anderson and Trueman* is the most important parasites of the western honey bee, *Apis mellifera L*. The most widely currently used treatment uses formic acid (FA), but the understanding of its effects on *V*. *destructor* is limited. In order to understand the mechanism of action of FA, its effect on Varroa mites was investigated using proteomic analysis by liquid chromatography-mass spectrometry/mass spectrometry (LC-MS/MS). *V*. *destructor* was collected from honey bee colonies with natural mite infestation before and 24 h after the initiation of FA treatment and subjected to proteome analysis. A total of 2637 proteins were identified. Quantitative analysis of differentially expressed candidate proteins (fold change ≥ 1.5; p ≤ 0.05) revealed 205 differentially expressed proteins: 91 were induced and 114 repressed in the FA-treated group compared to the untreated control group. Impaired protein synthesis accompanied by increased protein and amino acid degradation suggest an imbalance in proteostasis. Signs of oxidative stress included significant dysregulation of candidate proteins of mitochondrial cellular respiration, increased endocytosis, and induction of heat shock proteins. Furthermore, an increased concentration of several candidate proteins associated with detoxification was observed. These results suggest dysregulated cellular respiration triggered by FA treatment as well as an increase in cellular defense mechanisms, including induced heat shock proteins and detoxification enzymes.

## Introduction

The ectoparasite *Varroa destructor Anderson and Trueman* was transferred from the eastern honey bee, *Apis cerana F*., to the western honey bee, *Apis mellifera L*., in the early 19th century. Since then, it has been considered one of the greatest threats to the western honey bee [1]. In addition to habitat loss and pesticide use, it contributes significantly to the periodically high winter losses [2–4].

The Varroa mites reproduce in sealed brood cells [5] and cause considerable damage to their host. The damage is caused in two ways: first, the mite sucks on fatty tissue and haemolymph of adult and juvenile bees [6,7], but in addition, various pathogens such as viruses are transmitted into the bee [8–10]. Infestation is associated with reduction of weight and life span, malformation and weakening of the individual due to the loss of substance and possible suppression of the immune system by mite saliva [11–14]. The loss of individuals leads to the weakening of the entire colony as a superorganism through loss of colony functionality [15] and the ability to perform thermoregulation in winter [16], and finally the loss of untreated colonies within one to three years [17,18].

Earlier treatments with lipophilic hydrophobic substances such as fluvalinate and coumaphos had the disadvantage of accumulating mainly in beeswax and causing development of resistance in the mite [19,20], and thus the development of treatment with organic acids became a major therapy option. Among the organic acids formic acid (FA) has the advantage that it also penetrates the sealed cells and is still effective [21–23]. FA damages both the phoretic and reproductive stage of the mite [24]. According to our current knowledge, its application does not lead to resistance of mites, and shows only minor residue problems in bee products [22,25]. These facts, and because FA is approved as a natural active ingredient in organic beekeeping, make FA particularly advantageous over synthetic acaricides and lead to its use worldwide. The main disadvantage of the treatment is the high variability in its effectiveness: the amount of FA evaporated and thus the efficiency of the treatment varies greatly depending on external factors. These include primarily the ambient temperature and humidity, but also the strength of the bee colony, the occurrence of brood and the type of application used in the hive [3,26,27]. In addition, it also can cause damage on the bees [28–30]. There are several options for optimization, meaning increased varroacide action and little side effects, like online tools that are mostly based on the weather forecast since inappropriate treatment or unsuitable climatic conditions frequently lead to honey bee damage, such as increased mortality of queen, brood and freshly hatched workers [3,30,31].

Although extensive research has been conducted on the application of FA [26,30,32], surprisingly little is known about the mechanisms of FA damage and the molecular response in mites. Generally, it is suggested that FA binds to cytochrome c oxidase and thereby inhibits the mitochondrial electron transport chain [33–35] causing the mite to die. As a consequence, cellular respiration is thought to be inhibited and the body becomes acidotic [30]. Molecular biological studies to prove the actual harmful effect of FA in Varroa mites have not yet been implemented. In order to identify the actual processes going on in mites the assessment of the global proteome seems appropriate because in reaction to the treatment it is expected that changes occur that can be related to involved molecular pathways.

The aim of this project is to identify the molecular response of *V*. *destructor* to FA exposure. For this purpose, LC-MS/MS analysis was performed to identify regulated metabolic pathways and target structures of FA. These data may not only help to adapt and optimize FA treatment, but also to better control the parasite in the future through the discovery and exploitation of new cellular targets.

## Material and methods

### Formic acid treatment and sampling

*V*. *destructor* was obtained in two field trials conducted in 2018 and 2019, each from August to September, in the apiary of the Institute of Veterinary Biochemistry of the Department of Veterinary Medicine of the Free University of Berlin (latitude: 52.42898 °N, longitude: 13.23762 °E). In each year, four colonies of Western honey bee *A*. *mellifera carnica* were used. These were kept in Segeberger hives made of polystyrene, consisting of two boxes, a lid and a walkable bottom with bottom grid and showed moderate natural mite infestation with an average of 2.9 mites/day (2018) respectively 3 mites/day (2019). Besides that, all colonies of the apiary did not show any symptoms of disease and the absence of American foulbrood (*Paenibacillus larvae*) was confirmed by a health certificate.

In 2018, the average temperature recorded during the treatment period was 21 °C and precipitation averaged 12.2 liters per square meter. In the 2019 season, the average daily temperature during the treatment period was 20.3°C and the average precipitation was 28.2 liters per square meter.

All experimental colonies received treatment during the experiment with 200 ml of 60% FA ad us. vet. (Serumwerk Bernburg AG, Bernburg, Germany) through a Nassenheider evaporator universal R applicator (Joachim Weiland Werkzeugbau GmbH & Co. KG, Hoppegarten, Germany). An average of 10 ml of FA evaporated per day and hive. The applicators were removed from the colonies after approximately 10 days, after FA had completely evaporated.

Viable adult female V. destructor mites in reproductive and phoretic stages from the above-mentioned honey bee colonies were used for the analyses. As a control, mites were collected immediately before the start of the experiment (0 hours, control). 24 hours after the beginning of FA treatment, mites were collected again (24 hours, treatment). For this purpose, individual cells were opened and reproductive mites were removed from the capped brood cells using brushes and forceps. Phoretic mites were collected either directly from the bodies of adult honey bees with a fine bristle brush or from the surface of the adhesive board placed under the frames to determine mite infestation. It was ensured that all mites were alive. In addition, only clearly adult female mites were used for further analysis, which was evident from their size and brownish-red coloration. Mites of both stages collected from all experimental colonies were transferred to 1.5 ml microcentrifuge tubes and stored at -80°C until further processing.

A total of eight biological replicates were used for further analyses, four in the control group and four in the treatment group (0 and 24 hours). One biological replicate consisted of a pool of 40 individuals, i.e., a total of 320 individual mites were used for this experiment. In each pool, mites from reproductive and phoretic phases were mixed from all experimental colonies sampled in both years, 2018 and 2019. Pooling mites from different locations within the hive (inside the cell, on the adult bees, and on the sticky board) was intended to balance potential differences in exposed FA concentrations to assess the effect of FA on all stages of the mite in the hive and thus reflect conditions as close to practical conditions as possible. Furthermore, by conducting the studies in two different years, seasonal differences, such as weather fluctuations, nectar and pollen supply, should be compensated.

### Protein isolation

The lysis buffer (urea 9 M, chaps 2%) was prepared by adding 10 mg dithiothreitol (DTT), 5 μl phenylmethylsulfonyl fluoride (PMSF) and 1 μl protease inhibitor cocktail (Merck Biosciences GmbH, Protease Inhibitor Set III Cat.Nr.539134) per 1 ml buffer.

40 Varroa mites were homogenized in 500 μl ice-cooled lysis buffer using a tissue homogenizer (FastPrep FP120, Qbiogene Inc, Illkirch, France) and 1.4 mm ceramic beads in homogenization tubes (MP Biomedicals, Heidelberg, Germany). Four homogenization cycles were conducted for 15 s at 6 m/s, resting one minute on ice between each cycle to avoid heat-damaging of the samples. After homogenization the samples were put on ice for 1 h. Every 20 min a thorough mixing was performed by means of a vortexer (IKA Labortechnik, Staufen im Breisgau, Germany). The samples were then centrifuged at 16,200 x g for 10 min at 4 °C. The upper aqueous phase was carefully transferred to a new 1.5 ml microcentrifuge tube and the total protein content of each sample was determined using the Pierce^™^ 660 nm Protein Assay (Thermo Scientific, Karlsruhe, Germany) according to the manufacturer’s instructions by use of a BSA standard.

### Proteome analysis using LC-MS/MS

Protein samples were used for one-dimensional (1D) gel electrophoresis. For each sample 38 μg protein was mixed with 5x Lane Marker Reducing Sample Buffer (Thermo Scientific, USA) and incubated under shaking at 1400 rpm, 90 °C for 3 min. Samples were loaded on sodium dodecyl sulfate gels (4% stacking gel and 12% separating gel). Electrophoresis was performed at 10 mA per gel. Proteins were stained by colloidal Coomassie Brilliant Blue G-250 (Roth, Kassel, Germany). Entire lanes were cut into gel pieces for each sample and an in-gel tryptic cleavage was performed. Finally, samples were desalted and purified by SOLAμ (Thermo Scientific, USA) as suggested by the manufacturer. After evaporation peptides were resuspended in 15 μL 0.1% FA.

For each LC-MS/MS run 5 μL of total peptide lysate was injected into nanoHPLC (UltiMate 3000 RSLCnano, Dionex, Thermo Fisher Scientific). Peptides were trapped on a C18-reverse phase trapping column (μPAC^™^ Trapping column, Pharmafluidics, Belgium), followed by separation on a C18-reverse phase analytical column (50 cm μPAC^™^ column, Pharmafluidics). For separation a two-step gradient was applied as previously described [36]. Mass spectrometric analysis of eluted peptides was performed on a Q Exactive HF mass spectrometer (Thermo Fisher Scientific, Waltham, MA, USA) coupled with a TriVersa NanoMate (Advion, UK) source in LC chip coupling mode. A data dependent MS/MS measurement was performed in positive mode with settings previously described [36].

MS data processing was performed using Proteome Discoverer (v.2.2, Thermo Fischer Scientific, USA) with Sequest HT search engine against all listed "*V*.*destructor*" proteins of the NCBI-RefSeq database (status as of 25.09.2019; filtered to no redundancy, 20.089 protein entries). Search settings were set to trypsin (Full, max. missed cleavage sites: 2, precursor mass tolerance: 10 ppm, fragment mass tolerance: 0.05 Da. Carbamidomethylation of cysteines was specified as a fixed modification, oxidation of methionines and N-terminal acetylation as dynamic modifications). False discovery rates (FDR) were determined using Percolator (Käll et al. 2007). Proteins were considered as identified when at least one unique peptide was identified and the overall protein FDR was ≤ 0.05. Proteins were quantified based on the intensities of the top three identified peptides. The data was log_2_ transformed and median normalized.

### Result processing

Statistical analysis of the log_2_-transformed FCs was performed in R-3.5.0. To unravel significant (p-value ≤ 0.05) changes compared to control the Student’s t-test was performed for analytes that were quantified in at least three of four biological replicates over all the treatments. The obtained p-values were Benjamini & Hochberg adjusted.

For all identified differentially expressed proteins (DEP) between control and treatment group the log_2_ fold changes were calculated. For the quantitative analysis only DEPs with a fold change of at least 2 or above and a p-value of at least 0.05 or below were used, which were found in at least three of four samples (control and treatment group).

A principal component analysis (PCA) was performed to describe differences in the general distribution patterns of protein expression profiles between the control and treatment group.

The significantly regulated protein sequences were reannotated using eggnog mapper [37] to allow functional annotation based on the Kyoto Encyclopedia of Genes and Genomes (KEGG) database [38] and evaluation of their accumulation in specific pathways.

## Results

### Differentially expressed proteins after formic acid treatment

A total of 2637 proteins were identified. An average of 2272 proteins could be relatively quantified. The high number of proteins provides a solid base for the analysis of potential changes caused by FA treatment.

**Fig 1:**
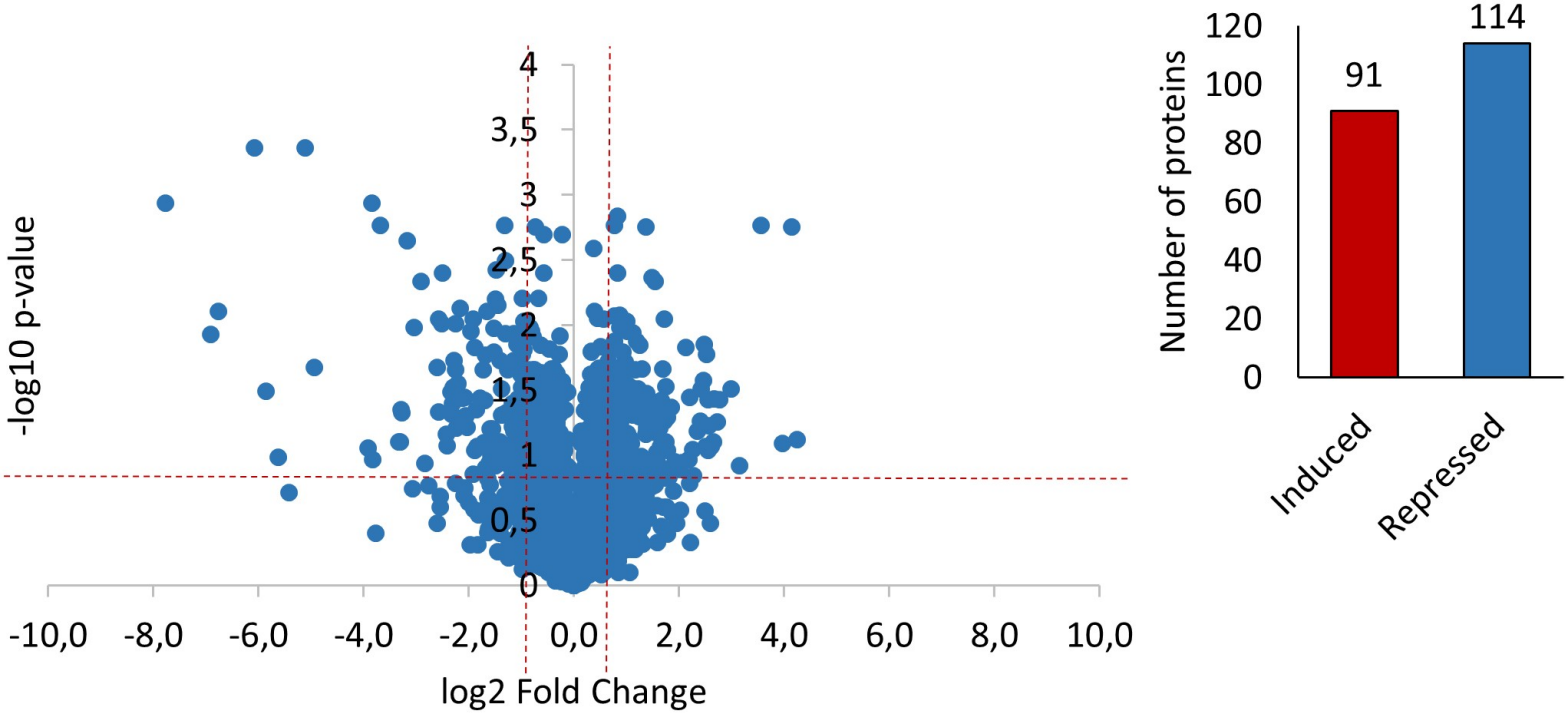
Volcano-Plot analysis and number of DEPs in FA-treated Varroa mites. Volcano plot for differential protein expression: Scattered points represent DEPs. The DEPs between the untreated control and treatment groups are plotted on the x-axis (log_2_ scale) and the statistical significance (p ≤ 0.05) on the y-axis (-log10 scale). The dotted lines show the fold changes above or below a 1.5-fold up or down control (values to the right and left of the vertical lines) and the statistical significance of p ≤ 0.05 (values above the horizontal line). Thus, the points in the upper left quadrant are proteins significantly down-regulated after FA treatment, and points in the upper right quadrant are proteins significantly up-regulated after FA treatment. A total of 115 proteins were significantly differentially expressed. 91 of them were up-regulated (at least 1.5-fold up-regulation, p ≤ 0.05) and 114 were down-regulated (at least 1.5-fold down-regulation, p ≤ 0.05).

A comparison of global protein expression between the FA-treated group and untreated control cohort revealed 205 differentially expressed proteins that met the selected criteria of fold change ≥ 1.5 and p-value ≤ 0.05. These included 91 increased and 114 decreased proteins in the FA-treated group compared to the untreated control group (Fig 1). An overview of all DEPs identified is documented in the appendix (S1 Table).

**Fig 2:**
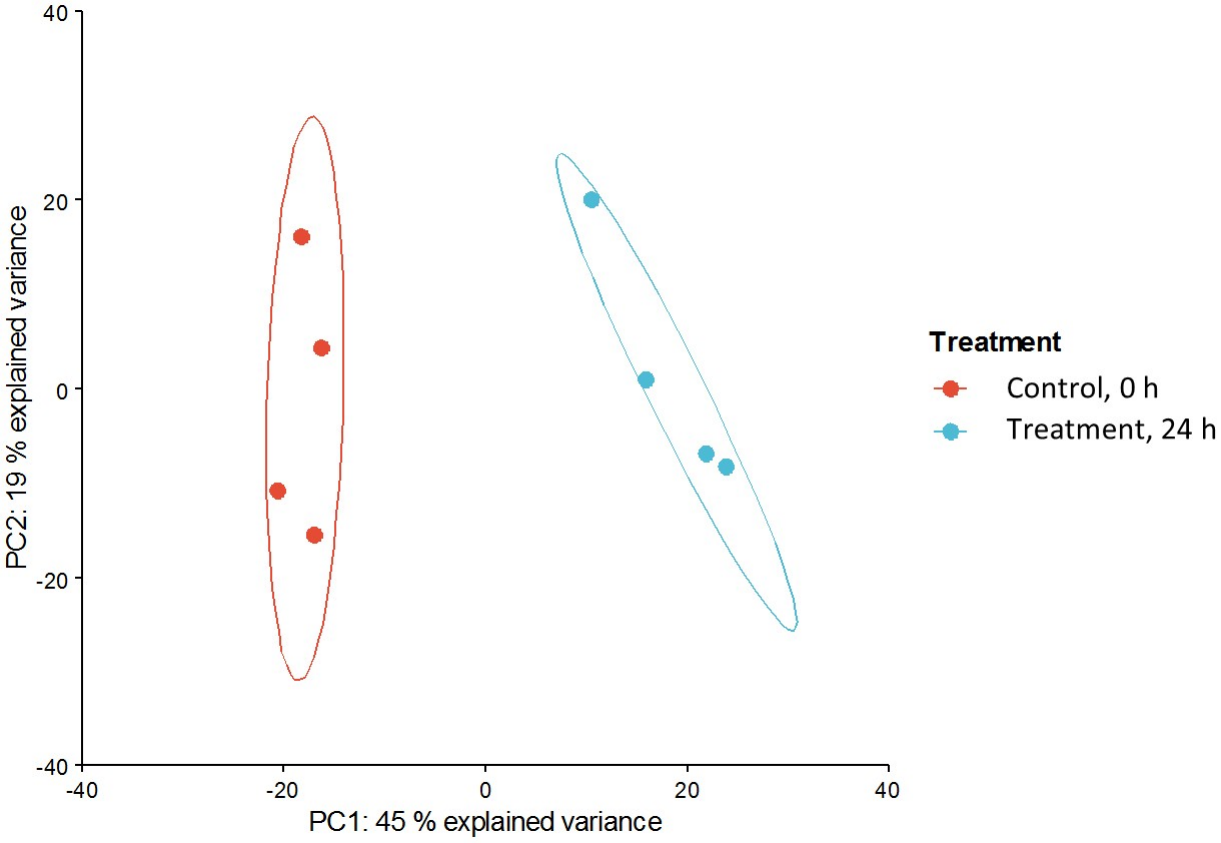
PCA to investigate the variance in the complete data matrix. Replicates of the control group are shown in red, those of the treatment group in blue.

In order to prove the reproducibility of the group separation based on proteomics data a PCA was performed. (Fig 2). The PCA clearly describes the alterations in protein expression at different times of FA exposure and obviously separates the control and treatment samples into specific groups. The first principal component (PC1) described 45% and PC2 19% of the variance between the control and treatment groups. These data show that the group separation was obtained with high reproducibility.

### KEGG pathway analysis

First, the DEPs were characterised with KEGG annotations to investigate the pathways in which they are potentially involved. A total of 101 DEPs could be assigned a KEGG ID, which corresponds to 49.72% of the significantly differentially expressed mite proteins. This is caused mainly by the uncomplete assignment of the *V*. *destructor* genome. The KEGG IDs of the proteins were then assigned to the pathways. In total, a participation of DEPs in 175 signaling pathways could be shown. A total of six signaling pathways were potentially involved in the molecular response to FA exposure, with an adjusted p-value < 0.05 and pathway coverage of more than one protein. The number of proteins in each significant pathway was maximum 5 and the pathway coverage maximum 21.74%. Significantly enriched pathways included amino acid and fatty acid degradation, Ras signalling pathway, and endocytosis. A table of all DEPs to which a KEGG number could be assigned is provided in the Appendix (S2 Table).

**Fig 3:**
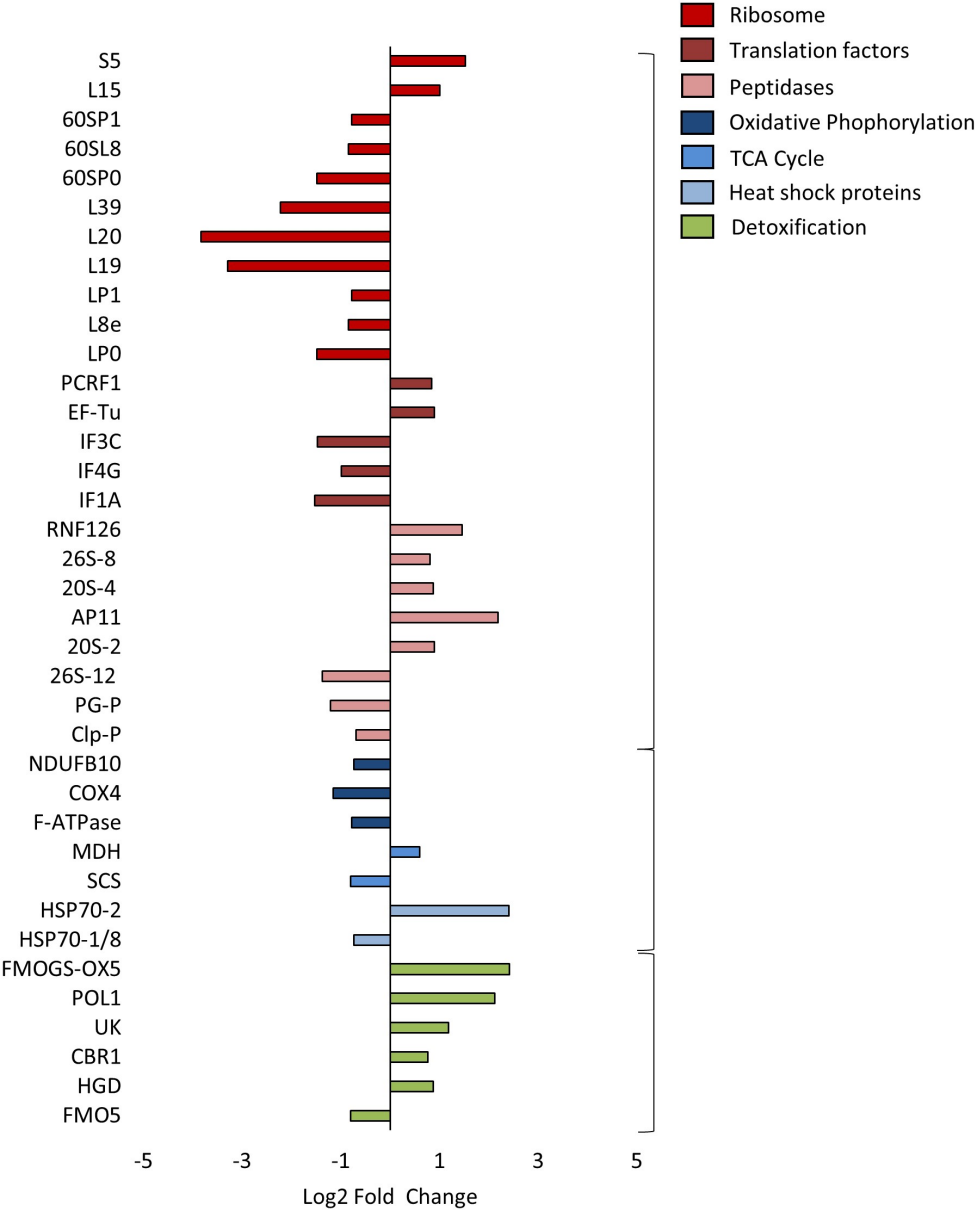
Profile of DEPs in 24 h FA-treated mites versus untreated control group. The shown DEPs are at least 1.5-fold and significantly (p-value ≤ 0.05) regulated. A classification was made according to involvement in proteostasis (red-based colours), cell respiration respectively oxidative stress (blue-based colours) and detoxification (green).

In addition, other mite proteins were significantly differentially expressed without accumulating in a signalling pathway. These included proteins that are associated with proteostasis. These included proteins associated with the ribosome, which were present in mostly reduced concentrations (*Large Subunit Ribosomal Protein LP39* (log_2_ fold change: -2.23; t(6): -5,4; p: 0.03), *60S Acidic Ribosomal Protein P0-like* (log_2_ fold change: -1.49; t(6): -11,84; p: 0.01)). Translation factors, found dysregulated (*Translation Initiation Factor 1A* (log_2_ fold change: -1.52; t(6): -6,68; p: 0.02), *Translation Initiation Factor 4G* (log_2_ fold change: -0.99; t(6): -4,39; p: 0.05)) were predominantly present in lower concentrations after FA exposure. In contrast, the protein concentration of structures involved in protein degradation was mainly induced (*20S Proteasome Subunit Alpha 4* (log_2_ Fold Change: 0.87; t(6): 8,77; p: 0.01), *E3 Ubiquitin-Protein Ligase RNF126-like* (log_2_ Fold Change: 1.46; t(6): 4,04; p: 0.05)). Furthermore, in most cases there was a significant decrease in the concentration of proteins associated with cell respiration (*Cytochrome C Oxidase Subunit 4* (log_2_ Fold Change: -1.15; t(6): -6,38; p: 0.02), *Succinyl-CoA Synthetase Beta Subunit* (log_2_Fold Change: -0.80; t(6): -5,5; p: 0.03)). Among the proteins with induced concentration were the enzymes associated with xenobiotic biodegradation and metabolism, according to the KEGG annotation, *Homogentisate-1*,*2-Dioxygenase* (log_2_ Fold Change: 0.87; t(6): 5,28; p: 0.03), *Carbonyl Reductase 1* (log_2_ Fold Change: 0.77; t(6): 4,59; p: 0.04) and *Flavin-Containing Monooxygenase (FMO) GS-OX5*-like (log_2_ Fold Change: 2.42; t(6): 5,21; p: 0.03). A selection of DEPs is shown in Fig 3 according to their involvement in different categories of cellular function.

## Discussion

Few recent publications addressed Varroa proteomics studies. The study by Erban et al (2015) presented the proteome of pathogens in *V*.*destructor*. In our study, a total of 2637 proteins were identified, of which an average of 2272 proteins could be relatively quantified. This is within the range of mite proteins previously identified by McAfee et al. [39] and Surlis et al. [40]. McAfee et al (2017) [39] used an intensity-based label-free quantification method to identify a total of 3,102 proteins, including 1,433 differently expressed proteins across different developmental stages (egg, protonymph, deuteronymph and adult). Surlis et al [40] identified a total of 3757 peptides, representing 650 proteins with two or more peptides. A very recent study performed a proteome analysis on chemosensory organs of reproductive and phoretic Varroa mites and identified a total of 958 Varroa proteins [41].

To our knowledge, the influence of the varroacide FA on protein expression in the Varroa mite has not been investigated in previous studies. Our holistic protein expression study was the first to demonstrate the effects of a common FA treatment, showing corresponding protein changes in Varroa mites.

### Differentially expressed proteins and affected pathways after formic acid treatment

**Fig 4:**
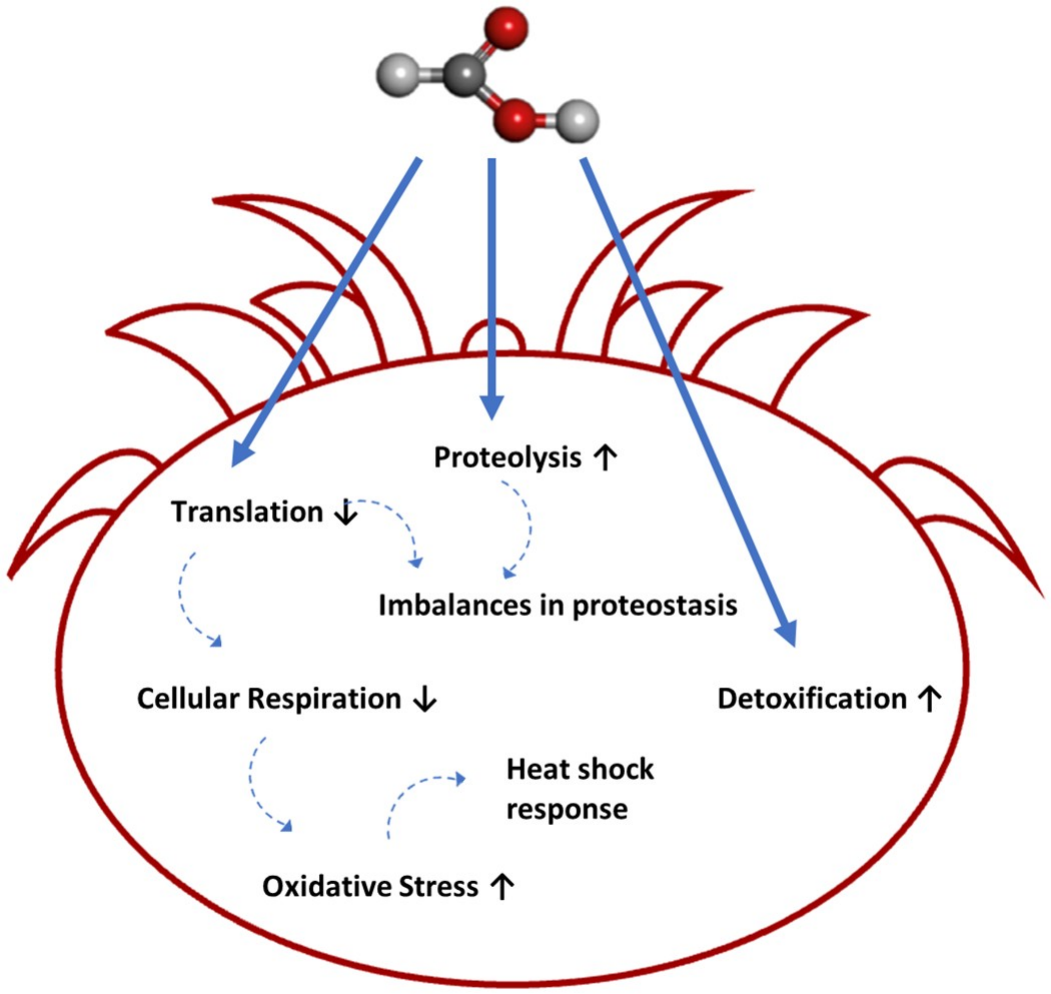
Schematic representation of mechanism of action of FA. Blue solid arrows show FA influence on various processes. Blue dotted arrows indicate an interaction of the processes. Arrows pointing up or down next to the process definition indicate whether the interaction is positive or negative.

Since the Varroa mite is not a model organism and its protein functions are mostly unknown, only limited pathway coverages could be identified in most cases. Our analysis revealed a total of six significantly enriched pathways in which more than one protein could be identified. To discuss the effects of FA on the proteome most comprehensively, additional significantly differentially expressed proteins that did not show significant pathway accumulation will also be discussed in the following (Fig 4).

The analysis showed a significant dysregulation of proteins of mitochondrial cell respiration in FA-exposed mites. The significantly lower concentration of *Succinyl-CoA Synthetase* indicates an inhibition of the tricarboxylic acid (TCA) cycle, which provides intermediates for the biosynthesis of various macromolecules as well as energy and electron acceptors (flavin adenine dinucleotide (FADH), nicotinamide adenine dinucleotide (NADH)) as the central metabolic centre of aerobic organisms. A dysfunctional TCA cycle can therefore lead to various functional disorders and cell pathologies due to the lack of substrates for synthesis processes and the subsequent inhibition of the respiratory chain.

Furthermore, the results after exposure to FA showed a significantly reduced quantity of several enzymes associated with the respiratory chain (*NADH Dehydrogenase* (complex I), *Cytochrome C Oxidase* (complex IV), *F-type H+-transporting ATPase* (complex V)). A number of studies have already demonstrated an inhibition of the mitochondrial electron transport chain through binding of complex IV by FA [33–35]. Impairments of respiratory metabolism are also evident in honey bees: under laboratory conditions, high concentrations of FA in the air inhibit oxygen uptake in young and adult bees [30]. The study of Zerin et al. [42] additionally demonstrated an inhibition of complex I by formaldehyde, a metabolite of FA, in neuroblastoma cells. A mitochondrial dysfunction triggered by FA would have far-reaching consequences: the mitochondrial energy metabolism produces more than 90% of the cell energy as ATP. The resulting ATP deficiency would have negative effects on important ATP-consuming metabolic pathways. These include exercise, active transport and biosynthesis [43]. Disorders often manifest as psychomotor disorders, acidosis and hypoglycaemia [30,44,45]. Inhibition of the respiratory chain also leads to accumulation of NADH [46], which further blocks the TCA cycle.

A disruption of the mitochondrial respiratory chain is also associated with an increased production of reactive oxygen species (ROS) [47,48]. These lead to uncontrolled oxidation of DNA, carbohydrates, proteins and lipids through so-called oxidative stress [49]. Insects usually react to this with symptoms of aging, which can finally result in cell death [50]. An indication of oxidative stress is the significant increase in the concentration of the *Heat shock 70kDa protein* (*HSP70*) in FA-treated mites. Heat shock proteins (HSP), also known as stress proteins, protect the cells under disadvantageous conditions from influences such as heat, nutrient deficiency and oxidative stress [51–53].

The expression of *HSP70* under stress conditions has already been described in insects [54]. For instance, FA treatment in honey bees resulted in increased HSP70 levels after treatment with high (85%) and low (30%) concentrations compared to the untreated control [55]. Additionally, hypoxia leads to the expression of several HSP genes in *Sarcophaga crassipalpis* and *Drosophila melanogaster*, including *HSP70* [56,57] and also a direct influence of FA on HSP expression in *Saccharomyces cerevisiae* has already been demonstrated (Lee, Park et al. 2010). Consequently, also in this case FA treatment with potential inhibition of cellular respiration could have led to oxidative stress and, in response, to overexpression of *HSP70* in the mites.

Another response to oxidative stress could be the significantly enriched *endocytosis signaling pathway*. In a review by López-Hernández et al. [58] induction of endocytosis in response to oxidative stress was reported in several contexts. However, according to the authors, further studies are needed to further elucidate the effects of ROS on endocytosis and the underlying molecular mechanisms.

Furthermore, the proteome analysis of the FA-treated Varroa mites indicated an imbalance in proteostasis due to a reduced protein synthesis with increased protein degradation. A similar effect of FA on protein biosynthesis has already been demonstrated in yeast cells as a result of a downregulation of ribosome biogenesis [59]. In honey bees, FA lowers protein concentration, has a time-dependent effect on protease activity, and overall leads to suppression of the proteolytic system on the body surface of bees [60]. In our case, the limited protein biosynthesis capacity of FA-treated mites was observed on the one hand by a general tendency of reduction of ribosome-associated proteins and on the other hand by a predominantly repressed expression of various translation factors. At the same time an induction of proteasome-associated proteins could be shown. Ribosomes are essential for protein synthesis and cell survival. Inhibition of ribosome synthesis (ribosomal stress) would lead to inhibition of cell growth and cell division with resulting developmental disorders and aging processes [61]. The proteasome is a highly organized protease complex responsible for regulated proteolysis during cell death and development in eukaryotic cells [62]. An increased activation of the proteasome with simultaneous inhibition of the biosynthesis processes would result in a rapid total protein loss. This means a significant loss of mass in the entire organism [63] and could explain a damaging effect of FA on the Varroa mites. One reason for the imbalance of proteostasis may be nutrient deficiency, in particular the lack of essential amino acids, leads to inhibition of ribosome synthesis [64] and at the same time, due to increased activity of the proteasome, to an increased overall degradation of cell proteins to ensure the supply of the organism with amino acids that are important for gluconeogenesis and energy production [65]. The FA treatment could lead to a weakening of the mites with subsequent reduced food intake, which would influence the activity of protein build-up and breakdown.

In addition to nutrient deficiency, cellular stressors such as hypoxia may also lead to inhibition of ribosome synthesis [66] and activation of proteasome-mediated protein degradation [67,68]. This effect could have occurred in FA-treated mites due to mitochondrial dysfunction with a limited respiratory chain (i.e., inhibition of cellular respiration). The oxygen radicals produced by inhibition of the respiratory chain can also lead to an increased occurrence of damaged proteins, which would be selectively eliminated by an increased activity of the proteasome [63].

Proteins of branched-chain amino acid metabolism (BBAA) and lipid metabolism accumulated in the significantly enriched pathways. As described above, FA treatment could lead to reduced food intake by the weakened mites, which would induce BCAA and fatty acid degradation. BBAAs serve as substrates for protein synthesis and play a critical role in determining the structures of globular proteins [69]. They also stimulate protein synthesis and inhibit proteolysis [70]. Thus, their degradation could further support an imbalance in proteostasis, as described above.

The proteome analysis further revealed an increased concentration of several candidate proteins associated with detoxification. Among these is *Carbonyl Reductase 1*, which is an important non-P450 pathway for the metabolism of both endogenous substances and xenobiotics [71,72]. It belongs to the group of NADPH-dependent short-chain reductases with broad substrate specificity [73] and plays an important role in drug metabolism and in protecting the organism from potentially harmful carbonyls and quinones [74].

Another detoxification enzyme induced after 24 h FA exposure is the *Homogentisate 1*,*2-Dioxygenase* (*HGD*), which is involved in the degradation of the amino acids tyrosine and phenylalanine [75]. In a feeding trial of honey bees an induction of this enzyme was demonstrated after feeding with honey [76]. A study in *Exophiala lecanii-corni* investigating the degradation mechanism of volatile organic compounds (VOCs), which includes FA, describes the involvement of *HGD* in the metabolism of ethylbenzene [77].

The induction of the *Peroxidase-like isoform X1*, an antioxidant enzyme, is a further indication of oxidative stress, which can be triggered by a potential inhibition of the respiratory chain. When the capacity of the antioxidative systems of the cell is exceeded, increased ROS production by damage to macromolecules triggers cell death. This could be one of the damaging mechanisms of action of FA on Varroa mites.

Interestingly, an increase in the concentration of the *FMO GS-OX5-like* could be observed in FA-exposed mites. The FMO GS-OXs are considered to be a specific enzyme group of cruciferous plants (Brassicaceae) and catalyze the first structural modification step in the synthesis of aliphatic glucosinolates [78,79]. In contrast to this and also in contrast to the results of a distinct FMO5 transcript analysis [80], the results of the protein expression analysis showed a significantly reduced concentration of another FMO, *FMO5*. FMOs represent a family of enzymes that metabolise xenobiotic compounds in the so-called phase I [81–83]. In the Varroa mite, the above mentioned enzymes have not been described in connection with FA detoxification so far, but have been associated with the development of resistance to certain chemical pesticides in lepidopteran species and in the Varroa mite [40,84].

In the future, a functional study to determine enzyme activity should provide information on the involvement of the above-mentioned enzymes in FA metabolism.

## Conclusion

To our knowledge, this is the first study to characterize FA-dependent protein changes in the Varroa mite. In summary, the study of protein concentrations provided information on the primary site of action of FA (components of the respiratory chain), its molecular effects in the organism (dysregulation of proteostasis, oxidative stress), and detoxification and defense mechanisms, which presumably include heat shock proteins and detoxification enzymes.

However, this study only includes effects that occur at an early stage of treatment (24h). Further studies should therefore investigate the expression of selected proteins over time during a standard FA treatment for an average of ten days.

However, a conclusive evidence for the association of the regulated proteins with FA exposure has not yet been established by this study. Ultimately, this can only be achieved by elucidating the functional correlation, since the function of an enzyme is not characterized by the abundance of the associated protein alone. Therefore, the function of the identified proteins should be further investigated in the future in order to increase our knowledge of the cellular structures that FA or other drugs might target. This will enable new treatment strategies against the pathogen *V*.*destructor* based on unique cellular targets and/ or pathways.

## Supporting information

S1 Table Overview of all DEPs. (PDF)

S2 Table Overview of all DEPs to which a KEGG number could be assigned. (PDF)

## References

[1] Nazzi F, Le Conte Y. Ecology of Varroa destructor, the major ectoparasite of the western honey bee, Apis mellifera. Annual Review of Entomology. 2016;61:417–32. doi: 10.1146/annurev-ento-010715-023731 26667378

[2] Le Conte Y, Ellis M, Ritter W. Varroa mites and honey bee health: can Varroa explain part of the colony losses? Apidologie. 2010;41(3):353–63.

[3] Rosenkranz P, Aumeier P, Ziegelmann B. Biology and control of Varroa destructor. Journal of invertebrate pathology. 2010;103:S96–S119. doi: 10.1016/j.jip.2009.07.016 19909970

[4] Genersch E, Von Der Ohe W, Kaatz H, Schroeder A, Otten C, Büchler R, et al. The German bee monitoring project: a long term study to understand periodically high winter losses of honey bee colonies. Apidologie. 2010;41(3):332–52.

[5] Martin SJ. Ontogenesis of the mite Varroa jacobsoni Oud. in worker brood of the honeybee Apis mellifera L. under natural conditions. Experimental & applied acarology. 1994;18(2):87–100.

[6] Sammataro D, Gerson U, Needham G. Parasitic mites of honey bees: life history, implications, and impact. Annual review of entomology. 2000;45(1):519–48. 10.1146/annurev.ento.45.1.519 10761588

[7] Ramsey SD, Ochoa R, Bauchan G, Gulbronson C, Mowery JD, Cohen A, et al. Varroa destructor feeds primarily on honey bee fat body tissue and not hemolymph. Proceedings of the National Academy of Sciences. 2019;116(5):1792–801. doi: 10.1073/pnas.1818371116 30647116 PMC6358713

[8] Ball B, editor association of Varroa jacobsoni with virus diseases of honey bees. Varroa jacobsoni Oud affecting honey bees: present status and needs: proceedings of a meeting of the EC Experts’ Group, Wageningen, 7–9 February 1983/edited by R Cavalloro; 1983: Rotterdam: Published for the Commission of the European Communities by AA.

[9] Chen Y, Pettis JS, Evans JD, Kramer M, Feldlaufer MF. Transmission of Kashmir bee virus by the ectoparasitic mite Varroa destructor. Apidologie. 2004;35(4):441–8.

[10] Tantillo G, Bottaro M, Di Pinto A, Martella V, Di Pinto P, Terio V. Virus infections of honeybees Apis Mellifera. Italian journal of food safety. 2015;4(3). doi: 10.4081/ijfs.2015.5364 27800411 PMC5076640

[11] De Jong D, De Jong P, Goncalves L. Weight loss and other damage to developing worker honeybees from infestation with Varroa jacobsoni. Journal of apicultural research. 1982;21(3):165–7.

[12] Duay P, De Jong D, Engels W. Weight loss in drone pupae (Apis mellifera) multiply infested by Varroa destructor mites. Apidologie. 2003;34(1):61–5.

[13] Dainat B, Evans JD, Chen YP, Gauthier L, Neumann P. Dead or alive: deformed wing virus and Varroa destructor reduce the life span of winter honeybees. Appl Environ Microbiol. 2012;78(4):981–7. doi: 10.1128/AEM.06537-11 22179240 PMC3273028

[14] Garedew A, Schmolz E, Lamprecht I. The energy and nutritional demand of the parasitic life of the mite Varroa destructor. Apidologie. 2004;35(4):419–30.

[15] FILIPOVIC M. Social minimum required for particular functions of worker bees (Apis mellifica L.). 1972.

[16] Schäfer MO, Ritter W, Pettis JS, Neumann P. Concurrent parasitism alters thermoregulation in honey bee (Hymenoptera: Apidae) winter clusters. Annals of the Entomological Society of America. 2011;104(3):476–82.

[17] Fries I, Imdorf A, Rosenkranz P. Survival of mite infested (Varroa destructor) honey bee (Apis mellifera) colonies in a Nordic climate. Apidologie. 2006;37(5):564–70.

[18] Le Conte Y, De Vaublanc G, Crauser D, Jeanne F, Rousselle J-C, Bécard J-M. Honey bee colonies that have survived Varroa destructor. Apidologie. 2007;38(6):566–72.

[19] Wallner K. Varroacides and their residues in bee products. Apidologie. 1999;30(2–3):235–48.

[20] Milani N. The resistance of Varroa jacobsoni Oud. to acaricides. Apidologie. 1999;30(2–3):229–34.

[21] Program IIftUNO. Formic Acid. Technical Evaluation Report 2011.

[22] Amrine JW Jr, Noel R. Formic acid fumigator for controlling varroa mites in honey bee hives. International Journal of Acarology. 2006;32(2):115–24.

[23] Devi S. Different methods for the management of Varroa mite (Varroa destructor) in honey bee colony. 2019.

[24] Fries I. Treatment of sealed honey bee brood with formic acid for control of Varroa jacobsoni. American bee journal (USA). 1991.

[25] Imdorf A, Charrière J-D, Kilchenmann V, Bogdanov S, Fluri P. Alternative strategy in central Europe for the control of Varroa destructor in honey bee colonies. Apiacta. 2003;38(3):258–78.

[26] Calderone NW. Evaluation of formic acid and a thymol-based blend of natural products for the fall control of Varroa jacobsoni (Acari: Varroidae) in colonies of Apis mellifera (Hymenoptera: Apidae). Journal of Economic Entomology. 1999;92(2):253–60.

[27] Imdorf A, Charrière J-D, Rosenkranz P. Varroa control with formic acid. FAIR CT97-3686. 1999:24.

[28] Gregorc A, Pogacnik A, Bowen ID. Cell death in honeybee (Apis mellifera) larvae treated with oxalic or formic acid. Apidologie. 2004;35(5):453–60.

[29] Ostermann DJ, Currie RW. Effect of formic acid formulations on honey bee (Hymenoptera: Apidae) colonies and influence of colony and ambient conditions on formic acid concentration in the hive. Journal of economic Entomology. 2004;97(5):1500–8. doi: 10.1603/0022-0493-97.5.1500 15568335

[30] Bolli H, Bogdanov S, Imdorf A, Fluri P. Zur Wirkungsweise von Ameisensäure bei Varroa jacobsoni Oud und der Honigbiene (Apis mellifera L). Apidologie. 1993;24(1):51–7.

[31] Dietemann V, Pflugfelder J, Anderson D, Charrière J-D, Chejanovsky N, Dainat B, et al. Varroa destructor: research avenues towards sustainable control. Journal of Apicultural Research. 2012;51(1):125–32.

[32] Satta A, Floris I, Eguaras M, Cabras P, Garau VL, Melis M. Formic acid-based treatments for control of Varroa destructor in a Mediterranean area. Journal of economic entomology. 2005;98(2):267–73. doi: 10.1093/jee/98.2.267 15889712

[33] Nicholls P. Formate as an inhibitor of cytochrome c oxidase. Biochemical and biophysical research communications. 1975;67(2):610–6. doi: 10.1016/0006-291x(75)90856-6 1020

[34] Keyhani J, Keyhani E. EPR study of the effect of formate on cytochrome c oxidase. Biochemical and biophysical research communications. 1980;92(1):327–33. doi: 10.1016/0006-291x(80)91556-9 6243938

[35] Liesivuori J, Savolainen, Heikki. Methanol and formic acid toxicity: biochemical mechanisms. Pharmacology & toxicology. 1991;69(3):157–63. doi: 10.1111/j.1600-0773.1991.tb01290.x 1665561

[36] Haange S-B, Jehmlich N, Hoffmann M, Weber K, Lehmann Jr, von Bergen M, et al. Disease development is accompanied by changes in bacterial protein abundance and functions in a refined model of dextran sulfate sodium (DSS)-induced colitis. Journal of proteome research. 2019;18(4):1774–86. doi: 10.1021/acs.jproteome.8b00974 30767541

[37] Huerta-Cepas J, Forslund K, Coelho LP, Szklarczyk D, Jensen LJ, Von Mering C, et al. Fast genome-wide functional annotation through orthology assignment by eggNOG-mapper. Molecular biology and evolution. 2017;34(8):2115–22. doi: 10.1093/molbev/msx148 28460117 PMC5850834

[38] Kanehisa M, Goto S. KEGG: kyoto encyclopedia of genes and genomes. Nucleic acids research. 2000;28(1):27–30. doi: 10.1093/nar/28.1.27 10592173 PMC102409

[39] McAfee A, Chan QW, Evans J, Foster LJ. A Varroa destructor protein atlas reveals molecular underpinnings of developmental transitions and sexual differentiation. Molecular & Cellular Proteomics. 2017;16(12):2125–37. doi: 10.1074/mcp.RA117.000104 28867676 PMC5724176

[40] Surlis C, Carolan JC, Coffey MF, Kavanagh K. Proteomic analysis of Bayvarol^®^ resistance mechanisms in the honey bee parasite Varroa destructor. Journal of Apicultural Research. 2016;55(1):49–64.

[41] Iovinella I, McAfee A, Mastrobuoni G, Kempa S, Foster LJ, Pelosi P, et al. Proteomic analysis of chemosensory organs in the honey bee parasite Varroa destructor: a comprehensive examination of the potential carriers for semiochemicals. Journal of proteomics. 2018;181:131–41. doi: 10.1016/j.jprot.2018.04.009 29653265

[42] Zerin T, Kim J-S, Gil H-W, Song H-Y, Hong S-Y. Effects of formaldehyde on mitochondrial dysfunction and apoptosis in SK-N-SH neuroblastoma cells. Cell biology and toxicology. 2015;31(6):261–72. doi: 10.1007/s10565-015-9309-6 26728267

[43] Berg JM, Tymoczko JL, Stryer L. Der Stoffwechsel: Konzepte und Grundmuster. Stryer Biochemie: Springer; 2013. p. 431–55.

[44] Blass J, Sheu R, Cedarbaum J. Energy metabolism in disorders of the nervous system. Revue neurologique. 1988;144(10):543–63. 2973643

[45] Reddi AS. Acid-Base Disorders: Clinical Evaluation and Management: Springer Nature; 2019. 317 p.

[46] Moreno-Domínguez A, Ortega-Sáenz P, Gao L, Colinas O, García-Flores P, Bonilla-Henao V, et al. Acute O2 sensing through HIF2α-dependent expression of atypical cytochrome oxidase subunits in arterial chemoreceptors. Science Signaling. 2020. doi: 10.1126/scisignal.aay9452 31848220

[47] Distelmaier F, Koopman WJH, van den Heuvel LP, Rodenburg RJ, Mayatepek E, Willems PHGM, et al. Mitochondrial complex I deficiency: from organelle dysfunction to clinical disease. Brain. 2009;132(4):833–42. doi: 10.1093/brain/awp058 19336460

[48] Schaffer WM, Bronnikova T. Peroxidase-ROS interactions. Nonlinear Dynamics. 2012;68(3):413–30.

[49] Sohal RS, Mockett RJ, Orr WC. Mechanisms of aging: an appraisal of the oxidative stress hypothesis. Free Radical Biology and Medicine. 2002;33(5):575–86. doi: 10.1016/s0891-5849(02)00886-9 12208343

[50] Fleming J, Reveillaud I, Niedzwiecki A. Role of oxidative stress in Drosophila aging. Mutation Research/DNAging. 1992;275(3–6):267–79. doi: 10.1016/0921-8734(92)90031-j 1383769

[51] Zügel U, Kaufmann SH. Role of heat shock proteins in protection from and pathogenesis of infectious diseases. Clinical Microbiology Reviews. 1999;12(1):19–39. doi: 10.1128/CMR.12.1.19 9880473 PMC88905

[52] Chappell TG, Welch WJ, Schlossman DM, Palter KB, Schlesinger MJ, Rothman JE. Uncoating ATPase is a member of the 70 kilodalton family of stress proteins. Cell. 1986;45(1):3–13. doi: 10.1016/0092-8674(86)90532-5 2937542

[53] Hightower LE. Heat shock, stress proteins, chaperones, and proteotoxicity. Cell. 1991;66(2):191–7. doi: 10.1016/0092-8674(91)90611-2 1855252

[54] King AM, MacRae TH. Insect heat shock proteins during stress and diapause. Annual review of entomology. 2015;60:59–75. doi: 10.1146/annurev-ento-011613-162107 25341107

[55] Gunes N, Aydın L, Belenli D, Hranitz JM, Mengilig S, Selova S. Stress responses of honey bees to organic acid and essential oil treatments against varroa mites. Journal of Apicultural Research. 2017;56(2):175–81.

[56] Azad P, Ryu J, Haddad GG. Distinct role of Hsp70 in Drosophila hemocytes during severe hypoxia. Free Radical Biology and Medicine. 2011;51(2):530–8. doi: 10.1016/j.freeradbiomed.2011.05.005 21616137 PMC3138732

[57] Michaud MR, Teets NM, Peyton JT, Blobner BM, Denlinger DL. Heat shock response to hypoxia and its attenuation during recovery in the flesh fly, Sarcophaga crassipalpis. Journal of insect physiology. 2011;57(1):203–10. doi: 10.1016/j.jinsphys.2010.11.007 21075112

[58] López-Hernández T, Haucke V, Maritzen T. Endocytosis in the adaptation to cellular stress. Cell Stress. 2020;4(10):230. doi: 10.15698/cst2020.10.232 33024932 PMC7520666

[59] Lee S-E, Park B-S, Yoon J-J. Proteomic Evaluation of Cellular Responses of S accharomyces cerevisiae to Formic Acid Stress. Mycobiology. 2010;38(4):302–9. doi: 10.4489/MYCO.2010.38.4.302 23956670 PMC3741523

[60] Strachecka AJ, Paleolog J, Borsuk G, Olszewski K. The influence of formic acid on the body surface proteolytic system at different developmental stages in Apis mellifera L. workers. Journal of Apicultural Research. 2012;51(3):252–62.

[61] Pestov DG, Strezoska Ž, Lau LF. Evidence of p53-dependent cross-talk between ribosome biogenesis and the cell cycle: effects of nucleolar protein Bop1 on G1/S transition. Molecular and cellular biology. 2001;21(13):4246–55. doi: 10.1128/MCB.21.13.4246-4255.2001 11390653 PMC87085

[62] Tanaka K. The proteasome: overview of structure and functions. Proceedings of the Japan Academy, Series B. 2009;85(1):12–36. doi: 10.2183/pjab.85.12 19145068 PMC3524306

[63] Lecker SH, Solomon V, Mitch WE, Goldberg AL. Muscle protein breakdown and the critical role of the ubiquitin-proteasome pathway in normal and disease states. The Journal of nutrition. 1999;129(1):227S–37S. doi: 10.1093/jn/129.1.227S 9915905

[64] Mayer C, Grummt I. Ribosome biogenesis and cell growth: mTOR coordinates transcription by all three classes of nuclear RNA polymerases. Oncogene. 2006;25(48):6384–91. doi: 10.1038/sj.onc.1209883 17041624

[65] Medina R, Wing SS, Goldberg AL. Increase in levels of polyubiquitin and proteasome mRNA in skeletal muscle during starvation and denervation atrophy. Biochemical Journal. 1995;307(3):631–7. doi: 10.1042/bj3070631 7741690 PMC1136697

[66] Mekhail K, Rivero-Lopez L, Khacho M, Lee S. Restriction of rRNA synthesis by VHL maintains energy equilibrium under hypoxia. Cell Cycle. 2006;5(20):2401–13. doi: 10.4161/cc.5.20.3387 17102617

[67] Gomes-Marcondes MCC, Tisdale MJ. Induction of protein catabolism and the ubiquitin-proteasome pathway by mild oxidative stress. Cancer letters. 2002;180(1):69–74. doi: 10.1016/s0304-3835(02)00006-x 11911972

[68] de Theije CC, Langen RC, Lamers WH, Gosker HR, Schols AM, Koehler SE. Differential sensitivity of oxidative and glycolytic muscles to hypoxia-induced muscle atrophy. Journal of Applied Physiology. 2015;118(2):200–11. doi: 10.1152/japplphysiol.00624.2014 25429096

[69] Brosnan JT, Brosnan ME. Branched-chain amino acids: enzyme and substrate regulation. The Journal of nutrition. 2006;136(1):207S–11S. doi: 10.1093/jn/136.1.207S 16365084

[70] Holeček M. Branched-chain amino acids in health and disease: metabolism, alterations in blood plasma, and as supplements. Nutrition & metabolism. 2018;15(1):1–12. 29755574 10.1186/s12986-018-0271-1 PMC5934885

[71] Malátková P, Wsol V. Carbonyl reduction pathways in drug metabolism. Drug metabolism reviews. 2014;46(1):96–123. doi: 10.3109/03602532.2013.853078 24171394

[72] Ramsden D, Smith D, Arenas R, Frederick K, Cerny MA. Identification and characterization of a selective human carbonyl reductase 1 substrate. Drug Metabolism and Disposition. 2018;46(10):1434–40. doi: 10.1124/dmd.118.082487 30068520

[73] Bateman RL, Rauh D, Tavshanjian B, Shokat KM. Human carbonyl reductase 1 is an S-nitrosoglutathione reductase. Journal of Biological Chemistry. 2008;283(51):35756–62. doi: 10.1074/jbc.M807125200 18826943 PMC2602912

[74] Boušová I, Skálová L, Souček P, Matoušková P. The modulation of carbonyl reductase 1 by polyphenols. Drug metabolism reviews. 2015;47(4):520–33. doi: 10.3109/03602532.2015.1089885 26415702

[75] Schmidt SR, Müller CR, Kress W. Murine liver homogentisate 1, 2-dioxygenase: Purification to homogeneity and novel biochemical properties. European journal of biochemistry. 1995;228(2):425–30. 7705358

[76] Wheeler MM, Robinson GE. Diet-dependent gene expression in honey bees: honey vs. sucrose or high fructose corn syrup. Scientific reports. 2014;4:5726. doi: 10.1038/srep05726 25034029 PMC4103092

[77] Gunsch CK, Cheng Q, Kinney KA, Szaniszlo PJ, Whitman CP. Identification of a homogentisate-1, 2-dioxygenase gene in the fungus Exophiala lecanii-corni: analysis and implications. Applied microbiology and biotechnology. 2005;68(3):405–11. doi: 10.1007/s00253-005-1899-0 15731901

[78] Li J, Hansen BG, Ober JA, Kliebenstein DJ, Halkier BA. Subclade of flavin-monooxygenases involved in aliphatic glucosinolate biosynthesis. Plant Physiology. 2008;148(3):1721–33. doi: 10.1104/pp.108.125757 18799661 PMC2577257

[79] Kong W, Li J, Yu Q, Cang W, Xu R, Wang Y, et al. Two novel flavin-containing monooxygenases involved in biosynthesis of aliphatic glucosinolates. Frontiers in plant science. 2016;7:1292. doi: 10.3389/fpls.2016.01292 27621741 PMC5003058

[80] Genath A, Sharbati S, Buer B, Nauen R, Einspanier R. Comparative transcriptomics indicates endogenous differences in detoxification capacity after formic acid treatment between honey bees and varroa mites. Scientific reports. 2020;10(1):1–14. 33318550 10.1038/s41598-020-79057-9 PMC7736338

[81] Lawton M, Cashman J, Cresteil T, Dolphin C, Elfarra A, Hines R, et al. A nomenclature for the mammalian flavin-containing monooxygenase gene family based on amino acid sequence identities. Archives of Biochemistry and Biophysics. 1994;308(1):254–7. doi: 10.1006/abbi.1994.1035 8311461

[82] Sehlmeyer S, Wang L, Langel D, Heckel DG, Mohagheghi H, Petschenka G, et al. Flavin-dependent monooxygenases as a detoxification mechanism in insects: new insights from the arctiids (Lepidoptera). PLoS One. 2010;5(5):e10435. doi: 10.1371/journal.pone.0010435 20454663 PMC2862711

[83] Berenbaum MR, Johnson RM. Xenobiotic detoxification pathways in honey bees. Current opinion in insect science. 2015;10:51–8. doi: 10.1016/j.cois.2015.03.005 29588014

[84] Dawkar VV, Chikate YR, Lomate PR, Dholakia BB, Gupta VS, Giri AP. Molecular insights into resistance mechanisms of lepidopteran insect pests against toxicants. Journal of Proteome Research. 2013;12(11):4727–37. doi: 10.1021/pr400642p 24090158

