# Supplementary material for: Influence of formic acid treatment on the proteome of the ectoparasite Varroa destructor
Source: PLoS One. 2021 Oct 26;16(10):e0258845. doi: 10.1371/journal.pone.0258845 (PMC8547630; doi:10.1371/journal.pone.0258845)
Supplement: S1 Table — (PDF) [file pone.0258845.s001.pdf]

Supporting information S2

Table S1: Overview of all DEPs to which a KEGG number could be assigned.

Accession number	Description	KO_Main_role	KO_Sub_role	KO_Pathway
XP_022650517.1	succinyl-CoA synthetase beta subunit	09100 Metabolism	09101 Carbohydrate metabolism	00020 Citrate cycle (TCA cycle) [PATH:ko00020]
XP_022649886.1	malate dehydrogenase	09100 Metabolism	09101 Carbohydrate metabolism	00020 Citrate cycle (TCA cycle) [PATH:ko00020]
XP_022668059.1	fructose-bisphosphate aldolase, class I	09100 Metabolism	09101 Carbohydrate metabolism	00030 Pentose phosphate pathway [PATH:ko00030]
XP_022672765.1	aldehyde reductase	09100 Metabolism	09101 Carbohydrate metabolism	00040 Pentose and glucuronate interconversions [PATH:ko00040]
XP_022672765.1	aldehyde reductase	09100 Metabolism	09101 Carbohydrate metabolism	00051 Fructose and mannose metabolism [PATH:ko00051]
XP_022672765.1	aldehyde reductase	09100 Metabolism	09101 Carbohydrate metabolism	00052 Galactose metabolism [PATH:ko00052]
XP_022643858.1	palmitoyl-protein thioesterase	09100 Metabolism	09103 Lipid metabolism	00062 Fatty acid elongation [PATH:ko00062]
XP_022657098.1	butyryl-CoA dehydrogenase	09100 Metabolism	09103 Lipid metabolism	00071 Fatty acid degradation [PATH:ko00071]
XP_022670962.1	NADH dehydrogenase (ubiquinone) 1 beta subcomplex subunit 10	09100 Metabolism	09102 Energy metabolism	00190 Oxidative phosphorylation [PATH:ko00190]
XP_022653165.1	F-type H+-transporting ATPase subunit g	09100 Metabolism	09102 Energy metabolism	00190 Oxidative phosphorylation [PATH:ko00190]
XP_022655493.1	cytochrome c oxidase subunit 4	09100 Metabolism	09102 Energy metabolism	00190 Oxidative phosphorylation [PATH:ko00190]

XP_022646767.1	5'-nucleotidase	09100 Metabolism	09104 Nucleotide metabolism	00230 Purine metabolism [PATH:ko00230]
XP_022653817.1	adenylate kinase	09100 Metabolism	09104 Nucleotide metabolism	00230 Purine metabolism [PATH:ko00230]
XP_022671354.1	adenosine kinase	09100 Metabolism	09104 Nucleotide metabolism	00230 Purine metabolism [PATH:ko00230]
XP_022661544.1	uridine phosphorylase	09100 Metabolism	09104 Nucleotide metabolism	00240 Pyrimidine metabolism [PATH:ko00240]
XP_022649886.1	malate dehydrogenase	09100 Metabolism	09105 Amino acid metabolism	00270 Cysteine and methionine metabolism [PATH:ko00270]
XP_022663326.1	glutathione synthase	09100 Metabolism	09105 Amino acid metabolism	00270 Cysteine and methionine metabolism [PATH:ko00270]
XP_022657098.1	butyryl-CoA dehydrogenase	09100 Metabolism	09105 Amino acid metabolism	00280 Valine, leucine and isoleucine degradation [PATH:ko00280]
XP_022657571.1	alpha-aminoadipic semialdehyde synthase	09100 Metabolism	09105 Amino acid metabolism	00310 Lysine degradation [PATH:ko00310]
XP_022649728.1	homogentisate 1,2-dioxygenase	09100 Metabolism	09105 Amino acid metabolism	00350 Tyrosine metabolism [PATH:ko00350]
XP_022663334.1	beta-mannosidase	09100 Metabolism	09107 Glycan biosynthesis and metabolism	00511 Other glycan degradation [PATH:ko00511]
XP_022643968.1	alpha-L-fucosidase	09100 Metabolism	09107 Glycan biosynthesis and metabolism	00511 Other glycan degradation [PATH:ko00511]
XP_022666911.1	complement component 1 Q subcomponent-binding protein, mitochondrial	09100 Metabolism	09107 Glycan biosynthesis and metabolism	00536 Glycosaminoglycan binding proteins [BR:ko00536]

XP_022653730.1	cell division cycle protein 37	09100 Metabolism	09107 Glycan biosynthesis and metabolism	00536 Glycosaminoglycan binding proteins [BR:ko00536]
XP_022672765.1	aldehyde reductase	09100 Metabolism	09103 Lipid metabolism	00561 Glycerolipid metabolism [PATH:ko00561]
XP_022652546.1	multiple inositol-polyphosphate phosphatase / 2,3-bisphosphoglycerate 3-phosphatase	09100 Metabolism	09101 Carbohydrate metabolism	00562 Inositol phosphate metabolism [PATH:ko00562]
XP_022665344.1	phosphatidylinositol 4-kinase type 2	09100 Metabolism	09101 Carbohydrate metabolism	00562 Inositol phosphate metabolism [PATH:ko00562]
XP_022651093.1	calcium-independent phospholipase A2	09100 Metabolism	09103 Lipid metabolism	00564 Glycerophospholipid metabolism [PATH:ko00564]
XP_022662805.1	carbonyl reductase 1	09100 Metabolism	09103 Lipid metabolism	00590 Arachidonic acid metabolism [PATH:ko00590]
XP_022649886.1	malate dehydrogenase	09100 Metabolism	09101 Carbohydrate metabolism	00620 Pyruvate metabolism [PATH:ko00620]
XP_022649886.1	malate dehydrogenase	09100 Metabolism	09101 Carbohydrate metabolism	00630 Glyoxylate and dicarboxylate metabolism [PATH:ko00630]
XP_022650517.1	succinyl-CoA synthetase beta subunit	09100 Metabolism	09101 Carbohydrate metabolism	00640 Propanoate metabolism [PATH:ko00640]
XP_022649728.1	homogentisate 1,2-dioxygenase	09100 Metabolism	09111 Xenobiotics biodegradation and metabolism	00643 Styrene degradation [PATH:ko00643]
XP_022649886.1	malate dehydrogenase	09100 Metabolism	09102 Energy metabolism	00710 Carbon fixation in photosynthetic organisms [PATH:ko00710]

XP_022658062.1	low molecular weight phosphotyrosine protein phosphatase	09100 Metabolism	09108 Metabolism of cofactors and vitamins	00730 Thiamine metabolism [PATH:ko00730]
XP_022658062.1	low molecular weight phosphotyrosine protein phosphatase	09100 Metabolism	09108 Metabolism of cofactors and vitamins	00740 Riboflavin metabolism [PATH:ko00740]
XP_022646767.1	5'-nucleotidase	09100 Metabolism	09108 Metabolism of cofactors and vitamins	00760 Nicotinate and nicotinamide metabolism [PATH:ko00760]
XP_022662805.1	carbonyl reductase 1	09100 Metabolism	09111 Xenobiotics biodegradation and metabolism	00980 Metabolism of xenobiotics by cytochrome P450 [PATH:ko00980]
XP_022653664.1	dimethylaniline monooxygenase (N-oxide forming)	09100 Metabolism	09111 Xenobiotics biodegradation and metabolism	00982 Drug metabolism - cytochrome P450 [PATH:ko00982]
XP_022668567.1	uridine kinase	09100 Metabolism	09111 Xenobiotics biodegradation and metabolism	00983 Drug metabolism - other enzymes [PATH:ko00983]
XP_022650038.1	nuclear receptor-binding protein	09100 Metabolism	09112 Enzyme families	01001 Protein kinases [BR:ko01001]
XP_022659550.1	mitogen-activated protein kinase kinase 1	09100 Metabolism	09112 Enzyme families	01001 Protein kinases [BR:ko01001]
XP_022665584.1	eukaryotic translation initiation factor 2-alpha kinase 3	09100 Metabolism	09112 Enzyme families	01001 Protein kinases [BR:ko01001]
XP_022672122.1	20S proteasome subunit alpha 4	09100 Metabolism	09112 Enzyme families	01002 Peptidases [BR:ko01002]
XP_022646811.1	20S proteasome subunit alpha 2	09100 Metabolism	09112 Enzyme families	01002 Peptidases [BR:ko01002]

XP_022650620.1	ATP-dependent Clp protease, protease subunit	09100 Metabolism	09112 Enzyme families	01002 Peptidases [BR:ko01002]
XP_022664109.1	pyroglutamyl-peptidase	09100 Metabolism	09112 Enzyme families	01002 Peptidases [BR:ko01002]
XP_022666956.1	abhydrolase domain-containing protein 11	09100 Metabolism	09112 Enzyme families	01002 Peptidases [BR:ko01002]
XP_022643858.1	palmitoyl-protein thioesterase	09100 Metabolism	09103 Lipid metabolism	01004 Lipid biosynthesis proteins [BR:ko01004]
XP_022658062.1	low molecular weight phosphotyrosine protein phosphatase	09100 Metabolism	09112 Enzyme families	01009 Protein phosphatase and associated proteins [BR:ko01009]
XP_022643827.1	heat shock 70kDa protein 1/8	09100 Metabolism	09112 Enzyme families	01009 Protein phosphatase and associated proteins [BR:ko01009]
XP_022662509.1	immunoglobulin-binding protein 1	09100 Metabolism	09112 Enzyme families	01009 Protein phosphatase and associated proteins [BR:ko01009]
XP_022668453.1	meiosis arrest female protein 1	09100 Metabolism	09112 Enzyme families	01009 Protein phosphatase and associated proteins [BR:ko01009]
XP_022671280.1	protein phosphatase 1H	09100 Metabolism	09112 Enzyme families	01009 Protein phosphatase and associated proteins [BR:ko01009]
XP_022643827.1	heat shock 70kDa protein 1/8	09130 Environmental Information Processing	09131 Membrane transport	02000 Transporters [BR:ko02000]
XP_022653517.1	solute carrier family 25 (mitochondrial dicarboxylate transporter), member 10	09130 Environmental Information Processing	09131 Membrane transport	02000 Transporters [BR:ko02000]
XP_022654997.1	signal recognition particle subunit SRP72	09130 Environmental	09131 Membrane transport	02044 Secretion system [BR:ko02044]

		Information Processing		
XP_022656610.1	cellular nucleic acid-binding protein	09120 Genetic Information Processing	09121 Transcription	03000 Transcription factors [BR:ko03000]
XP_022669895.1	ribosome assembly protein 1	09120 Genetic Information Processing	09122 Translation	03008 Ribosome biogenesis in eukaryotes [PATH:ko03008]
XP_022643827.1	heat shock 70kDa protein 1/8	09120 Genetic Information Processing	09122 Translation	03009 Ribosome biogenesis [BR:ko03009]
XP_022647013.1	large subunit ribosomal protein LP1	09120 Genetic Information Processing	09122 Translation	03010 Ribosome [PATH:ko03010]
XP_022646833.1	large subunit ribosomal protein L8e	09120 Genetic Information Processing	09122 Translation	03010 Ribosome [PATH:ko03010]
XP_022646731.1	large subunit ribosomal protein LP0	09120 Genetic Information Processing	09122 Translation	03010 Ribosome [PATH:ko03010]
XP_022655300.1	large subunit ribosomal protein L19	09120 Genetic Information Processing	09122 Translation	03010 Ribosome [PATH:ko03010]
XP_022657123.1	large subunit ribosomal protein L15	09120 Genetic Information Processing	09122 Translation	03010 Ribosome [PATH:ko03010]
XP_022666258.1	large subunit ribosomal protein L20	09120 Genetic Information Processing	09122 Translation	03010 Ribosome [PATH:ko03010]
XP_022668708.1	small subunit ribosomal protein S5	09120 Genetic Information Processing	09122 Translation	03010 Ribosome [PATH:ko03010]
XP_022671489.1	large subunit ribosomal protein L39	09120 Genetic Information Processing	09122 Translation	03011 Ribosome [BR:ko03011]
XP_022656771.1	elongation factor Tu	09120 Genetic Information Processing	09122 Translation	03012 Translation factors [BR:ko03012]
XP_022657429.1	translation initiation factor 4G	09120 Genetic Information Processing	09122 Translation	03012 Translation factors [BR:ko03012]

XP_022670350.1	peptide chain release factor subunit 1	09120 Genetic Information Processing	09122 Translation	03012 Translation factors [BR:ko03012]
XP_022652849.1	translation initiation factor 1A	09120 Genetic Information Processing	09122 Translation	03012 Translation factors [BR:ko03012]
XP_022668871.1	translation initiation factor 3 subunit C	09120 Genetic Information Processing	09122 Translation	03012 Translation factors [BR:ko03012]
XP_022650419.1	mRNA export factor	09120 Genetic Information Processing	09122 Translation	03013 RNA transport [PATH:ko03013]
XP_022657475.1	small nuclear ribonucleoprotein B and B'	09120 Genetic Information Processing	09124 Replication and repair	03032 DNA replication proteins [BR:ko03032]
XP_022646545.1	microtubule-associated protein, RP/EB family	09120 Genetic Information Processing	09124 Replication and repair	03036 Chromosome and associated proteins [BR:ko03036]
XP_022667854.1	splicing factor, arginine/serine-rich 1	09120 Genetic Information Processing	09121 Transcription	03040 Spliceosome [PATH:ko03040]
XP_022672923.1	splicing factor U2AF 65 kDa subunit	09120 Genetic Information Processing	09121 Transcription	03040 Spliceosome [PATH:ko03040]
XP_022647212.1	calcium homeostasis endoplasmic reticulum protein	09120 Genetic Information Processing	09121 Transcription	03040 Spliceosome [PATH:ko03040]
XP_022660835.1	peptidyl-prolyl isomerase H (cyclophilin H)	09120 Genetic Information Processing	09121 Transcription	03040 Spliceosome [PATH:ko03040]
XP_022671297.1	splicing factor 3A subunit 2	09120 Genetic Information Processing	09121 Transcription	03040 Spliceosome [PATH:ko03040]
XP_022660180.1	26S proteasome regulatory subunit N5	09120 Genetic Information Processing	09123 Folding, sorting and degradation	03050 Proteasome [PATH:ko03050]
XP_022667558.1	signal peptidase complex subunit 2	09120 Genetic Information Processing	09123 Folding, sorting and degradation	03060 Protein export [PATH:ko03060]

XP_022666047.1	guanine nucleotide-binding protein G(I)/G(S)/G(T) subunit beta-1	09130 Environmental Information Processing	09132 Signal transduction	04011 MAPK signaling pathway - yeast [PATH:ko04011]
XP_022647416.1	Rab family, other	09130 Environmental Information Processing	09133 Signaling molecules and interaction	04031 GTP-binding proteins [BR:ko04031]
XP_022670068.1	COP9 signalosome complex subunit 2	09120 Genetic Information Processing	09123 Folding, sorting and degradation	04121 Ubiquitin system [BR:ko04121]
XP_022647036.1	vesicle transport protein SEC22	09120 Genetic Information Processing	09123 Folding, sorting and degradation	04130 SNARE interactions in vesicular transport [PATH:ko04130]
XP_022667770.1	adaptin ear-binding coat-associated protein 1/2	09120 Genetic Information Processing	09123 Folding, sorting and degradation	04131 Membrane trafficking [BR:ko04131]
XP_022668845.1	low-density lipoprotein receptor-related protein 4	09120 Genetic Information Processing	09123 Folding, sorting and degradation	04131 Membrane trafficking [BR:ko04131]
XP_022661869.1	endophilin-A	09120 Genetic Information Processing	09123 Folding, sorting and degradation	04131 Membrane trafficking [BR:ko04131]
XP_022653082.1	actin related protein 2/3 complex, subunit 2	09120 Genetic Information Processing	09123 Folding, sorting and degradation	04131 Membrane trafficking [BR:ko04131]
XP_022655171.1	coatomer subunit epsilon	09120 Genetic Information Processing	09123 Folding, sorting and degradation	04131 Membrane trafficking [BR:ko04131]
XP_022656533.1	stonin-1/2	09120 Genetic Information Processing	09123 Folding, sorting and degradation	04131 Membrane trafficking [BR:ko04131]
XP_022656732.1	F-BAR domain only protein	09120 Genetic Information Processing	09123 Folding, sorting and degradation	04131 Membrane trafficking [BR:ko04131]
XP_022643858.1	palmitoyl-protein thioesterase	09140 Cellular Processes	09141 Transport and catabolism	04142 Lysosome [PATH:ko04142]

XP_022661778.1	phytanoyl-CoA hydroxylase	09140 Cellular Processes	09141 Transport and catabolism	04146 Peroxisome [PATH:ko04146]
XP_022656771.1	elongation factor Tu	09140 Cellular Processes	09141 Transport and catabolism	04147 Exosome [BR:ko04147]
XP_022654170.1	dimethylargininase	09140 Cellular Processes	09141 Transport and catabolism	04147 Exosome [BR:ko04147]
XP_022667514.1	ankyrin	09150 Organismal Systems	09151 Immune system	04624 Toll and Imd signaling pathway [PATH:ko04624]
XP_022669814.1	NADH dehydrogenase (ubiquinone) 1 beta subcomplex subunit 8	09150 Organismal Systems	09156 Nervous system	04723 Retrograde endocannabinoid signaling [PATH:ko04723]
