# Supplementary material for: Influence of formic acid treatment on the proteome of the ectoparasite Varroa destructor
Source: PLoS One. 2021 Oct 26;16(10):e0258845. doi: 10.1371/journal.pone.0258845 (PMC8547630; doi:10.1371/journal.pone.0258845)
Supplement: S2 Table — (PDF) [file pone.0258845.s002.pdf]

Supporting information S1

Table S1: Overview of all DEPs. The total sample size was n = 8 (control n = 4, treatment n = 4).

Accession number	Fold-change	t	p	Description
XP_022662741.1	0,60	5,16	0,03	LETM1 domain-containing protein 1-like [Varroa destructor]
XP_022649886.1	0,60	5,28	0,03	protein lunapark-B-like isoform X1 [Varroa destructor]
XP_022661692.1	0,61	4,27	0,05	leukocyte elastase inhibitor-like [Varroa destructor]
XP_022670068.1	0,63	4,82	0,04	COP9 signalosome complex subunit 2-like [Varroa destructor]
XP_022656378.1	0,64	4,57	0,04	importin subunit alpha-7-like [Varroa destructor]
XP_022661693.1	0,65	6,36	0,02	ovalbumin-related protein X-like [Varroa destructor]
XP_022669247.1	0,66	6,08	0,02	uncharacterized protein LOC111253709 [Varroa destructor]
XP_022643858.1	0,68	4,41	0,05	palmitoyl-protein thioesterase 1-like [Varroa destructor]
XP_022643904.1	0,69	4,97	0,03	store-operated calcium entry-associated regulatory factor-like isoform X1 [Varroa destructor]
XP_022672161.1	0,70	6,82	0,02	U6 snRNA-associated Sm-like protein LSm1 [Varroa destructor]
XP_022645818.1	0,70	5,54	0,03	hypoxanthine-guanine phosphoribosyltransferase-like [Varroa destructor]
XP_022660285.1	0,72	4,17	0,05	trafficking protein particle complex subunit 3-like isoform X1 [Varroa destructor]
XP_022646767.1	0,73	4,46	0,04	uncharacterized protein LOC111244180 isoform X1 [Varroa destructor]
XP_022672765.1	0,76	5,79	0,02	1,5-anhydro-D-fructose reductase-like isoform X1 [Varroa destructor]
XP_022662313.1	0,77	15,25	0,00	phosphofurin acidic cluster sorting protein 2-like isoform X1 [Varroa destructor]
XP_022662805.1	0,77	4,59	0,04	carbonyl reductase [NADPH] 1-like [Varroa destructor]
XP_022652666.1	0,77	7,18	0,01	glucose dehydrogenase [FAD, quinone]-like [Varroa destructor]
XP_022656929.1	0,77	8,70	0,01	sarcosine dehydrogenase, mitochondrial-like isoform X3 [Varroa destructor]
XP_022646263.1	0,77	7,21	0,01	RNA-binding protein Rsf1-like [Varroa destructor]
XP_022667482.1	0,81	5,14	0,03	26S proteasome regulatory subunit 8 isoform X1 [Varroa destructor]

XP_022652658.1	0,82	4,73	0,04	SH3 domain-binding glutamic acid-rich protein homolog isoform X1 [Varroa destructor]
XP_022667854.1	0,83	16,47	0,00	serine/arginine-rich splicing factor 1-like isoform X2 [Varroa destructor]
XP_022650841.1	0,83	10,87	0,00	leukocyte elastase inhibitor-like isoform X1 [Varroa destructor]
XP_022663720.1	0,84	4,10	0,05	delta-aminolevulinic acid dehydratase-like isoform X4 [Varroa destructor]
XP_022670350.1	0,84	4,18	0,05	eukaryotic peptide chain release factor subunit 1 isoform X1 [Varroa destructor]
XP_022643399.1	0,86	6,08	0,02	disks large homolog 1-like isoform X1 [Varroa destructor]
XP_022645555.1	0,87	8,77	0,01	transmembrane protease serine 9-like [Varroa destructor]
XP_022672122.1	0,87	7,94	0,01	proteasome subunit alpha type-7-like [Varroa destructor]
XP_022649728.1	0,87	5,28	0,03	homogentisate 1,2-dioxygenase-like [Varroa destructor]
XP_022646811.1	0,90	4,90	0,04	proteasome subunit alpha type-2-like [Varroa destructor]
XP_022655212.1	0,90	5,59	0,03	uncharacterized protein LOC111247927 isoform X1 [Varroa destructor]
XP_022656771.1	0,90	4,56	0,04	uncharacterized protein LOC111248552 [Varroa destructor]
XP_022672923.1	0,90	4,73	0,04	splicing factor U2AF 50 kDa subunit-like [Varroa destructor]
XP_022657098.1	0,93	6,68	0,02	short-chain specific acyl-CoA dehydrogenase, mitochondrial-like [Varroa destructor]
XP_022657447.1	0,94	4,39	0,05	ELAV-like protein 1 [Varroa destructor]
XP_022649780.1	0,94	5,21	0,03	aladin-like [Varroa destructor]
XP_022649081.1	0,95	4,40	0,05	gamma-taxilin-like isoform X1 [Varroa destructor]
XP_022651110.1	0,97	6,33	0,02	NFU1 iron-sulfur cluster scaffold homolog, mitochondrial-like [Varroa destructor]
XP_022657060.1	0,99	4,29	0,05	39S ribosomal protein L46, mitochondrial-like [Varroa destructor]
XP_022653082.1	1,00	4,26	0,05	actin-related protein 2/3 complex subunit 2-like isoform X1 [Varroa destructor]
XP_022657397.1	1,00	8,26	0,01	ras-related protein Rab-14-like [Varroa destructor]
XP_022654261.1	1,00	7,83	0,01	protein-L-isoaspartate(D-aspartate) O-methyltransferase-like isoform X1 [Varroa destructor]
XP_022657123.1	1,01	4,33	0,05	39S ribosomal protein L15, mitochondrial-like isoform X1 [Varroa destructor]
XP_022672301.1	1,01	4,60	0,04	dual specificity protein phosphatase 3-like isoform X1 [Varroa destructor]

XP_022669216.1	1,02	7,71	0,01	protein-lysine methyltransferase METTL21D-like [Varroa destructor]
XP_022662509.1	1,05	4,54	0,04	immunoglobulin-binding protein 1b-like [Varroa destructor]
XP_022658493.1	1,06	5,95	0,02	uncharacterized protein LOC111249200 isoform X1 [Varroa destructor]
XP_022669895.1	1,07	5,34	0,03	elongation factor-like GTPase 1 isoform X1 [Varroa destructor]
XP_022657753.1	1,11	4,19	0,05	vitellogenin-3-like [Varroa destructor]
XP_022657153.1	1,12	7,63	0,01	diphosphoinositol polyphosphate phosphohydrolase 3-alpha-like isoform X1 [Varroa destructor]
XP_022666467.1	1,14	5,39	0,04	uncharacterized protein LOC111252579 isoform X2 [Varroa destructor]
XP_022650016.1	1,16	5,91	0,02	uncharacterized protein LOC111245657 isoform X1 [Varroa destructor]
XP_022643449.1	1,16	4,29	0,05	2,3-bisphosphoglycerate-independent phosphoglycerate mutase-like isoform X1 [Varroa destructor]
XP_022668567.1	1,19	7,24	0,01	uridine-cytidine kinase 2-B-like isoform X1 [Varroa destructor]
XP_022660127.1	1,22	5,19	0,03	histidine triad nucleotide-binding protein 3-like [Varroa destructor]
XP_022654402.1	1,23	4,83	0,04	protein canopy 4-like [Varroa destructor]
XP_022658327.1	1,24	4,11	0,05	N-acetylgalactosamine kinase-like isoform X1 [Varroa destructor]
XP_022650038.1	1,25	8,30	0,01	nuclear receptor-binding protein-like isoform X1 [Varroa destructor]
XP_022665584.1	1,29	6,97	0,02	eukaryotic translation initiation factor 2-alpha kinase 3-like isoform X1 [Varroa destructor]
XP_022660835.1	1,30	4,40	0,05	peptidyl-prolyl cis-trans isomerase H-like [Varroa destructor]
XP_022666496.1	1,37	13,73	0,00	lysine--tRNA ligase-like [Varroa destructor]
XP_022671624.1	1,38	5,03	0,03	transmembrane protein 62-like [Varroa destructor]
XP_022668104.1	1,41	4,60	0,04	GDP-L-fucose synthase-like [Varroa destructor]
XP_022664578.1	1,41	4,18	0,05	tetratricopeptide repeat protein 1-like isoform X1 [Varroa destructor]
XP_022647036.1	1,42	4,31	0,05	vesicle-trafficking protein SEC22b-B-like [Varroa destructor]
XP_022665998.1	1,46	4,04	0,05	E3 ubiquitin-protein ligase RNF126-like [Varroa destructor]
XP_022660963.1	1,49	10,57	0,00	integrin alpha-PS1-like isoform X1 [Varroa destructor]
XP_022668708.1	1,53	4,47	0,04	28S ribosomal protein S5, mitochondrial-like isoform X1 [Varroa destructor]

XP_022665344.1	1,55	10,29	0,00	phosphatidylinositol 4-kinase type 2-alpha-like isoform X1 [Varroa destructor]
XP_022665961.1	1,58	4,82	0,05	striatin-interacting protein 1 homolog isoform X1 [Varroa destructor]
XP_022670740.1	1,65	4,14	0,05	uncharacterized protein LOC111254308 [Varroa destructor]
XP_022671439.1	1,66	4,71	0,04	transport and Golgi organization protein 2 homolog isoform X1 [Varroa destructor]
XP_022657475.1	1,69	6,10	0,02	small nuclear ribonucleoprotein-associated protein B-like isoform X1 [Varroa destructor]
XP_022646008.1	1,71	8,43	0,01	uncharacterized protein LOC111243925 isoform X1 [Varroa destructor]
XP_022666617.1	1,71	4,03	0,05	ribosomal protein S6 kinase beta-1-like isoform X1 [Varroa destructor]
XP_022659092.1	1,74	5,35	0,03	uncharacterized protein LOC111249466 [Varroa destructor]
XP_022647764.1	1,78	4,08	0,05	farnesyl pyrophosphate synthase-like isoform X1 [Varroa destructor]
XP_022670575.1	1,84	4,49	0,04	haloacid dehalogenase-like hydrolase domain-containing protein 2 [Varroa destructor]
XP_022659202.1	2,12	6,92	0,01	peroxidase-like isoform X1 [Varroa destructor]
XP_022666956.1	2,19	4,89	0,04	protein ABHD11-like isoform X1 [Varroa destructor]
XP_022650723.1	2,41	4,01	0,05	heat shock 70 kDa protein cognate 2-like [Varroa destructor]
XP_022670306.1	2,42	5,21	0,03	flavin-containing monooxygenase FMO GS-OX5-like [Varroa destructor]
XP_022659550.1	2,47	5,58	0,03	dual specificity mitogen-activated protein kinase kinase 1-like isoform X1 [Varroa destructor]
XP_022668477.1	2,48	7,09	0,01	histone H3-like [Varroa destructor]
XP_022656533.1	2,52	6,57	0,02	protein stoned-B-like isoform X1 [Varroa destructor]
XP_022652126.1	2,55	4,74	0,04	NEDD8-activating enzyme E1 catalytic subunit-like [Varroa destructor]
XP_022647416.1	2,67	4,78	0,04	GTP-binding protein ypt2-like [Varroa destructor]
XP_022668453.1	2,76	4,75	0,04	uncharacterized protein LOC111253396 isoform X1 [Varroa destructor]
XP_022656732.1	2,98	5,17	0,03	F-BAR domain only protein 2-like [Varroa destructor]
XP_022661193.1	3,56	14,49	0,00	rab proteins geranylgeranyltransferase component A 1-like isoform X1 [Varroa destructor]

XP_022651093.1	4,15	14,09	0,00	85/88 kDa calcium-independent phospholipase A2-like isoform X1 [Varroa destructor]
XP_022656610.1	-7,78	-24,71	0,00	DNA-binding protein HEXBP-like isoform X1 [Varroa destructor]
XP_022644180.1	-6,91	-7,52	0,01	cold shock protein 2-like [Varroa destructor]
XP_022664914.1	-6,77	-15,33	0,01	leucine-rich repeat flightless-interacting protein 2-like isoform X1 [Varroa destructor]
XP_022657977.1	-6,08	-23,57	0,00	calcium uniporter protein, mitochondrial-like isoform X1 [Varroa destructor]
XP_022671297.1	-5,85	-5,11	0,03	splicing factor 3A subunit 2-like [Varroa destructor]
XP_022663667.1	-5,11	-35,45	0,00	tetratricopeptide repeat protein 37-like isoform X1 [Varroa destructor]
XP_022671280.1	-4,94	-6,18	0,02	protein phosphatase 1H-like [Varroa destructor]
XP_022666258.1	-3,84	-17,77	0,00	uncharacterized protein LOC111252501 isoform X1 [Varroa destructor]
XP_022650138.1	-3,69	-19,40	0,00	mitochondrial intermembrane space import and assembly protein 40-B-like [Varroa destructor]
XP_022655300.1	-3,29	-4,41	0,05	39S ribosomal protein L19, mitochondrial-like [Varroa destructor]
XP_022647212.1	-3,27	-4,28	0,05	calcium homeostasis endoplasmic reticulum protein-like isoform X1 [Varroa destructor]
XP_022657666.1	-3,17	-12,65	0,00	uncharacterized protein LOC111248875 [Varroa destructor]
XP_022668338.1	-3,04	-8,01	0,01	eukaryotic translation initiation factor 4H-like isoform X1 [Varroa destructor]
XP_022645744.1	-2,90	-10,37	0,00	uncharacterized protein F12A10.7-like [Varroa destructor]
XP_022653817.1	-2,60	-6,17	0,02	adenylate kinase-like [Varroa destructor]
XP_022661089.1	-2,58	-8,41	0,01	zinc finger protein on ecdysone puffs-like [Varroa destructor]
XP_022649584.1	-2,58	-4,32	0,05	cuticle protein 65-like [Varroa destructor]
XP_022658098.1	-2,52	-8,14	0,01	uncharacterized protein LOC111249053 isoform X1 [Varroa destructor]
XP_022654661.1	-2,51	-10,91	0,00	protein RCC2-like [Varroa destructor]
XP_022649578.1	-2,35	-5,08	0,03	general transcriptional corepressor trfA-like [Varroa destructor]
XP_022652629.1	-2,32	-4,22	0,05	stromal cell-derived factor 2-like [Varroa destructor]

XP_022643968.1	-2,31	-5,12	0,04	alpha-L-fucosidase-like [Varroa destructor]
XP_022653517.1	-2,29	-5,27	0,03	mitochondrial dicarboxylate carrier-like [Varroa destructor]
XP_022662891.1	-2,28	-6,38	0,02	natterin-4-like [Varroa destructor]
XP_022660857.1	-2,25	-5,94	0,02	glycine-rich RNA-binding protein 2-like [Varroa destructor]
XP_022645657.1	-2,25	-8,16	0,01	MIF-like protein mif-2 [Varroa destructor]
XP_022671489.1	-2,23	-5,40	0,03	39S ribosomal protein L39, mitochondrial-like isoform X1 [Varroa destructor]
XP_022647433.1	-2,20	-5,43	0,03	importin subunit alpha-4-like [Varroa destructor]
XP_022663994.1	-2,17	-9,17	0,01	digestive cysteine proteinase 2-like [Varroa destructor]
XP_022667558.1	-2,12	-4,82	0,04	signal peptidase complex subunit 2-like [Varroa destructor]
XP_022647162.1	-2,08	-4,85	0,04	uncharacterized protein LOC111244381 [Varroa destructor]
XP_022649083.1	-2,07	-4,15	0,05	uncharacterized protein LOC111245245 [Varroa destructor]
XP_022657637.1	-2,06	-4,11	0,05	oxysterol-binding protein-related protein 9-like isoform X1 [Varroa destructor]
XP_022652113.1	-1,97	-7,70	0,01	neurofilament medium polypeptide-like [Varroa destructor]
XP_022665444.1	-1,92	-8,52	0,01	NADH-cytochrome b5 reductase 2-like [Varroa destructor]
XP_022651535.1	-1,89	-6,91	0,01	uncharacterized protein LOC111246340 [Varroa destructor]
XP_022669632.1	-1,87	-4,41	0,05	uncharacterized protein LOC111253826 [Varroa destructor]
XP_022671354.1	-1,78	-4,82	0,04	adenosine kinase 2-like isoform X1 [Varroa destructor]
XP_022654442.1	-1,73	-5,93	0,02	rab5 GDP/GTP exchange factor-like isoform X1 [Varroa destructor]
XP_022661409.1	-1,70	-4,72	0,04	serine/threonine-protein kinase PAK 1-like isoform X1 [Varroa destructor]
XP_022654170.1	-1,69	-6,54	0,02	N(G),N(G)-dimethylarginine dimethylaminohydrolase 1-like isoform X1 [Varroa destructor]
XP_022652546.1	-1,66	-8,91	0,01	multiple inositol polyphosphate phosphatase 1-like isoform X1 [Varroa destructor]
XP_022666219.1	-1,53	-7,89	0,01	uncharacterized protein LOC111252488 [Varroa destructor]
XP_022652849.1	-1,52	-6,68	0,02	eukaryotic translation initiation factor 1A, Y-chromosomal-like isoform X1 [Varroa destructor]
XP_022646731.1	-1,49	-11,84	0,01	60S acidic ribosomal protein P0-like [Varroa destructor]
XP_022668871.1	-1,48	-11,21	0,00	eukaryotic translation initiation factor 3 subunit C-like [Varroa destructor]
XP_022661544.1	-1,45	-9,30	0,01	uridine phosphorylase 2-like [Varroa destructor]

XP_022649450.1	-1,41	-6,40	0,02	polycystic kidney disease protein 1-like 3 [Varroa destructor]
XP_022650419.1	-1,38	-5,22	0,03	mRNA export factor-like [Varroa destructor]
XP_022660180.1	-1,38	-4,17	0,05	26S proteasome non-ATPase regulatory subunit 12-like [Varroa destructor]
XP_022670985.1	-1,32	-14,91	0,00	uncharacterized protein LOC111254418 isoform X1 [Varroa destructor]
XP_022663326.1	-1,31	-11,66	0,00	glutathione synthetase-like [Varroa destructor]
XP_022666047.1	-1,30	-7,59	0,01	guanine nucleotide-binding protein G(I)/G(S)/G(T) subunit beta-1 [Varroa destructor]
XP_022658137.1	-1,29	-4,30	0,05	glycine-rich cell wall structural protein 1.8-like [Varroa destructor]
XP_022647563.1	-1,26	-5,97	0,02	syntaxin-like [Varroa destructor]
XP_022664109.1	-1,21	-4,13	0,05	pyroglutamyl-peptidase 1-like [Varroa destructor]
XP_022668765.1	-1,20	-4,26	0,05	coatomer subunit beta-like isoform X1 [Varroa destructor]
XP_022648612.1	-1,19	-4,15	0,05	coatomer subunit delta-like [Varroa destructor]
XP_022649597.1	-1,18	-4,38	0,05	uncharacterized protein LOC111245471 [Varroa destructor]
XP_022662250.1	-1,17	-4,60	0,04	spidroin-2-like isoform X1 [Varroa destructor]
XP_022655493.1	-1,15	-6,38	0,02	cytochrome c oxidase subunit 4 isoform 1, mitochondrial-like isoform X1 [Varroa destructor]
XP_022667852.1	-1,14	-7,57	0,01	serine/arginine-rich splicing factor 1A-like isoform X1 [Varroa destructor]
XP_022650946.1	-1,11	-6,22	0,02	uncharacterized protein LOC111246029 isoform X1 [Varroa destructor]
XP_022667676.1	-1,09	-7,07	0,01	dolichyl-diphosphooligosaccharide--protein glycosyltransferase 48 kDa subunit-like [Varroa destructor]
XP_022647380.1	-1,07	-4,80	0,04	phosphoribosyl pyrophosphate synthase-associated protein 2-like isoform X1 [Varroa destructor]
XP_022655171.1	-1,02	-4,87	0,04	coatomer subunit epsilon-like [Varroa destructor]
XP_022661221.1	-1,02	-5,75	0,02	hydroxymethylglutaryl-CoA synthase 1-like isoform X1 [Varroa destructor]
XP_022660387.1	-1,00	-5,28	0,03	mesencephalic astrocyte-derived neurotrophic factor homolog [Varroa destructor]
XP_022663924.1	-1,00	-4,49	0,04	uncharacterized protein LOC111251536 isoform X1 [Varroa destructor]
XP_022668304.1	-1,00	-7,36	0,01	uncharacterized protein LOC111253325 [Varroa destructor]

XP_022657429.1	-0,99	-4,39	0,05	eukaryotic translation initiation factor 4 gamma 2-like [Varroa destructor]
XP_022658062.1	-0,99	-6,57	0,02	low molecular weight phosphotyrosine protein phosphatase-like [Varroa destructor]
XP_022653489.1	-0,98	-9,66	0,01	cingulin-like isoform X1 [Varroa destructor]
XP_022671762.1	-0,97	-6,80	0,02	ribose-5-phosphate isomerase-like isoform X1 [Varroa destructor]
XP_022665777.1	-0,96	-4,09	0,05	translationally-controlled tumor protein homolog [Varroa destructor]
XP_022663978.1	-0,95	-8,29	0,01	natterin-3-like [Varroa destructor]
XP_022656302.1	-0,95	-6,80	0,02	delta(3,5)-Delta(2,4)-dienoyl-CoA isomerase, mitochondrial-like [Varroa destructor]
XP_022661869.1	-0,89	-4,51	0,04	endophilin-A-like isoform X2 [Varroa destructor]
XP_022667303.1	-0,89	-4,02	0,05	ATPase family AAA domain-containing protein 3-B-like [Varroa destructor]
XP_022643440.1	-0,88	-4,23	0,05	soma ferritin-like [Varroa destructor]
XP_022646986.1	-0,87	-5,95	0,02	CDGSH iron-sulfur domain-containing protein 1-like isoform X1 [Varroa destructor]
XP_022661778.1	-0,86	-7,47	0,01	phytanoyl-CoA dioxygenase, peroxisomal-like [Varroa destructor]
XP_022646833.1	-0,84	-4,60	0,04	60S ribosomal protein L8-like [Varroa destructor]
XP_022654997.1	-0,84	-7,95	0,01	signal recognition particle subunit SRP72-like isoform X1 [Varroa destructor]
XP_022653664.1	-0,80	-7,74	0,01	dimethylaniline monooxygenase [N-oxide-forming] 5-like [Varroa destructor]
XP_022650517.1	-0,80	-5,50	0,03	succinate--CoA ligase [ADP-forming] subunit beta, mitochondrial-like isoform X1 [Varroa destructor]
XP_022668845.1	-0,79	-5,37	0,03	basement membrane-specific heparan sulfate proteoglycan core protein-like isoform X1 [Varroa destructor]
XP_022661063.1	-0,79	-4,87	0,04	T-complex protein 1 subunit delta-like [Varroa destructor]
XP_022666911.1	-0,78	-7,40	0,01	complement component 1 Q subcomponent-binding protein, mitochondrial-like isoform X1 [Varroa destructor]
XP_022647013.1	-0,78	-4,90	0,04	60S acidic ribosomal protein P1-like [Varroa destructor]
XP_022653165.1	-0,78	-5,18	0,03	ATP synthase subunit g, mitochondrial-like [Varroa destructor]
XP_022646476.1	-0,77	-5,93	0,02	acetylcholinesterase-1-like [Varroa destructor]
XP_022657571.1	-0,77	-4,13	0,05	alpha-aminoadipic semialdehyde synthase, mitochondrial-like isoform X1 [Varroa destructor]

XP_022669814.1	-0,75	-5,01	0,03	NADH dehydrogenase [ubiquinone] 1 beta subcomplex subunit 8, mitochondrial-like [Varroa destructor]
XP_022667770.1	-0,74	-5,93	0,02	NECAP-like protein CG9132 [Varroa destructor]
XP_022646545.1	-0,74	-4,75	0,04	microtubule-associated protein RP/EB family member 1-like [Varroa destructor]
XP_022663334.1	-0,74	-5,60	0,03	beta-mannosidase-like isoform X1 [Varroa destructor]
XP_022643827.1	-0,74	-4,61	0,04	heat shock-related 70 kDa protein 2-like [Varroa destructor]
XP_022661668.1	-0,73	-5,72	0,02	glycine-rich cell wall structural protein-like [Varroa destructor]
XP_022670962.1	-0,73	-13,85	0,00	NADH dehydrogenase [ubiquinone] 1 beta subcomplex subunit 10-like [Varroa destructor]
XP_022643642.1	-0,72	-4,22	0,05	probable imidazolonepropionase [Varroa destructor]
XP_022653730.1	-0,71	-5,03	0,03	hsp90 co-chaperone Cdc37-like [Varroa destructor]
XP_022656514.1	-0,70	-5,53	0,03	protein NDRG3-like isoform X1 [Varroa destructor]
XP_022653274.1	-0,70	-4,14	0,05	cuticle protein 14-like isoform X1 [Varroa destructor]
XP_022650620.1	-0,69	-4,71	0,04	uncharacterized protein LOC111245902 [Varroa destructor]
XP_022653613.1	-0,69	-4,16	0,05	nascent polypeptide-associated complex subunit alpha-like [Varroa destructor]
XP_022668059.1	-0,67	-9,64	0,01	fructose-bisphosphate aldolase A-like [Varroa destructor]
XP_022666309.1	-0,66	-4,34	0,05	neprilysin-1-like [Varroa destructor]
XP_022656777.1	-0,65	-4,43	0,04	probable serine/threonine-protein kinase clkA isoform X1 [Varroa destructor]
XP_022657427.1	-0,65	-5,40	0,03	uncharacterized protein LOC111248775 [Varroa destructor]
XP_022671579.1	-0,63	-7,05	0,01	sex-regulated protein janus-A-like isoform X3 [Varroa destructor]
XP_022649857.1	-0,63	-4,34	0,05	uncharacterized protein LOC111245586 [Varroa destructor]
XP_022667514.1	-0,62	-5,83	0,02	ankyrin-2-like isoform X1 [Varroa destructor]
XP_022666220.1	-0,60	-4,51	0,04	macrophage migration inhibitory factor-like [Varroa destructor]
